# The NLRP3 Inflammasome Increases Pulmonary Vascular Remodeling in Experimental Pulmonary Arterial Hypertension

**DOI:** 10.1002/pul2.70354

**Published:** 2026-07-17

**Authors:** Emmanouil Mavrogiannis, Rebeca Weldeghebreal, Iris R. Schilthuis, Zain K. Fal, Niels J. Kloosterhuis, Mirjam H. Koster, Wim Timens, Johannes M. Douwes, Rolf M. F. Berger, Marit Westerterp

**Affiliations:** ^1^ Center for Congenital Heart Diseases, Department of Pediatric Cardiology, Beatrix Children's Hospital University Medical Center Groningen, University of Groningen Groningen the Netherlands; ^2^ Department of Pediatrics University Medical Center Groningen, University of Groningen Groningen the Netherlands; ^3^ Department of Pathology and Medical Biology University Medical Center Groningen, University of Groningen Groningen the Netherlands

**Keywords:** AIM2 inflammasome, IL‐1β, NLRP3 inflammasome, pulmonary arterial hypertension

## Abstract

Pulmonary arterial hypertension (PAH) is a progressive vasculopathy leading to right‐sided heart failure. We have shown previously that the (NOD‐like‐receptor‐3) NLRP3 inflammasome is activated in end‐stage disease of the monocrotaline and aortocaval shunt (MCT/ACS) neointimal PAH rat model. The NLRP3 and absent‐in‐melanoma 2 (AIM2) inflammasomes mediate the secretion of interleukin (IL)‐1β and IL‐18. We investigated the role of the NLRP3 inflammasome in PAH rats and the presence of NLRP3 and AIM2 in PAH rat and pediatric patient lungs. For a natural history study, we assessed inflammasome activation by Western blot at different disease stages in the MCT/ACS neointimal PAH rat model. To assess the role of the inflammasome in PAH, we treated PAH rats with or without the NLRP3 inflammasome inhibitor MCC950 and assessed vascular remodeling employing hematoxylin and eosin staining on rat lungs. Lung sections from patients who had undergone lung transplantation were stained for NLRP3 and AIM2 using immunofluorescence. We found that inflammasome activation in lungs of PAH rats, reflected by cleaved caspase‐1, IL‐1β, and IL‐18, increased over time, reaching significance for cleaved caspase‐1 and IL‐18 in end‐stage PAH. The NLRP3 inflammasome inhibitor MCC950 suppressed vascular remodeling but not hemodynamic variables compared to control PAH rats. We detected NLRP3^+^ and AIM2^+^ cells in end‐stage pediatric PAH lungs. In conclusion, inflammasome activation increases over time in the MCT/ACS neointimal PAH rat model, and NLRP3 inflammasome activation increases vascular remodeling. NLRP3 and AIM2 positive cells are present in end‐stage pediatric patient PAH lungs, suggesting human relevance.

## 1 Introduction

Pulmonary arterial hypertension (PAH) is a rare, severe arteriopathy marked by its progressive nature, leading to right‐sided heart failure. Pediatric PAH has a more aggressive phenotype compared to the adult disease [1, 2]. In pediatric PAH, treatment options focus on vasodilation and right ventricular function, with heart and lung transplantation being the ultimate therapeutic option. PAH is characterized by a specific pattern of pulmonary vascular remodeling, including aberrant neointimal and plexiform lesions, both in the adult and pediatric population [3, 4].

The process of pulmonary remodeling in PAH, and the formation of neointimal lesions, is enhanced by endothelial inflammation, changes in the extracellular matrix, and perivascular leukocyte infiltration [5]. We previously found that the nucleotide‐binding domain, leucine‐rich‐containing family, pyrin domain containing 3 (NLRP3) inflammasome is activated in perivascular macrophages in the monocrotaline (MCT)/aortocaval shunt (ACS) rat model of neointimal PAH [6]. The NLRP3 inflammasome is the most well‐characterized inflammasome and regulates the secretion of pro‐inflammatory interleukin (IL)‐1β and IL‐18 [7]. Treatment with pirfenidone decreased NLRP3 inflammasome activation and ameliorated PAH disease progression in the MCT/ACS rat model of PAH [6]. However, in addition to its anti‐inflammatory properties, pirfenidone is mainly known for its anti‐fibrotic properties, and therefore, it remains to be assessed whether specific NLRP3 inhibition ameliorates PAH.

A role for the NLRP3 inflammasome in PAH has been corroborated by several studies. Studies in human adult PAH show high levels of plasma IL‐1β and IL‐18, the products of inflammasome activation [8, 9, 10, 11]. In more recent studies, Al‐Qazazi et al. found NLRP3 inflammasome activation in right ventricular macrophages of adult PAH patients and in right ventricular macrophages of the MCT and Sugen hypoxia rat models of PAH [12]. Moreover, the NLRP3 inflammasome inhibitor MCC950 improved right ventricular function in the MCT rat model of PH, assessed echocardiographically by tricuspid annular plane systolic excursion and cardiac index [12]. However, the effects of MCC950 on pulmonary vascular disease were not addressed in these studies, while our previous studies specifically found NLRP3 inflammasome activation in lung tissues of the MCT/ACS rat model of PAH [6]. Furthermore, in addition to the NLRP3 inflammasome, other inflammasomes may be activated in PAH. Previous studies have shown that neutrophil extracellular traps (NETs) accumulate in the lungs of patients with idiopathic PAH [13]. Via the release of their double‐stranded (ds)DNA, NETs may promote activation of the absent‐in‐melanoma 2 (AIM2) inflammasome [14].

We here investigated the natural history of inflammasome activation in the MCT/ACS rat model of neointimal PAH, with a focus on NLRP3 and AIM2 inflammasome activation in lung tissue. Next, we evaluated the effect of the NLRP3 inflammasome inhibitor MCC950 on PAH in the same rat model. Finally, we examined the presence of the NLRP3 and AIM2 inflammasomes in end‐stage PAH explant lung tissues from pediatric patients.

## 2 Methods

### 2.1 Natural History Study in the MCT/ACS Rat Model of Neointimal PAH

Lung tissue from a previously conducted study by van der Feen et al. was used [15]. In brief, 20 young adult male Wistar rats (200–250 g each) were randomly divided into four groups: a control group (control; *N* = 5), and three groups with induced PAH (PAH; *N* = 5 per group). For induction of PAH, on Day 1, animals were injected subcutaneously with 60 mg/kg body weight monocrotaline (MCT, PHL89251, Sigma‐Aldrich) dissolved in NaCl 0.9%. Controls were injected subcutaneously with NaCl 0.9%. On Day 7, PAH animals underwent aortocaval shunt surgery, and control animals underwent sham surgery, as previously described [6, 14, 15]. PAH animals were divided into three groups and sacrificed on Day 8 (T8, *N* = 5), reflecting the acute response to the hemodynamic loading on injured endothelium, Day 14 (T14, *N* = 5) to study the early‐stage flow‐associated PAH, and Day 28 (T28, *N* = 5) for late‐stage neointimal disease. Control animals were sacrificed on Day 28. On the final day of the experiment, animals were anesthetized with 5% isoflurane for induction and 2.5%–4% for maintenance, underwent closed‐chest right heart catheterization, and were subsequently sacrificed by exsanguination from the abdominal aorta. Subsequently, tissues were collected.

### 2.2 NLRP3 Inflammasome Inhibition With MCC950 in the MCT/ACS Rat Model of Neointimal PAH

Twenty‐four young adult male Wistar rats (200–250 g each) were randomly divided into three groups: a control group (control; *N* = 5), PAH (PAH; *N* = 10), and PAH with intervention (PAH + MCC950; *N* = 9). PAH was induced as described above. PAH animals received PBS vehicle intraperitoneally, while PAH + MCC950 animals received MCC950 sodium salt (Axon 4052, Axon Medchem, the Netherlands; 10 mg/kg bodyweight/day) dissolved in PBS intraperitoneally. Both groups received daily injections of PBS or PBS containing MCC950 from Day 8 up to Day 28. Controls did not receive daily intraperitoneal injections with PBS since our previous studies have shown that animals with and without PBS injections have the same characteristics regarding hemodynamics and vascular morphometry [15]. On Day 28, animals were anesthetized as described above, underwent closed‐chest right heart catheterization, and were subsequently sacrificed by exsanguination from the abdominal aorta, followed by tissue collection. One rat from the PAH + MCC950 group was found dead in its cage on Day 13 and therefore excluded from further analyses.

### 2.3 PAH Phenotype Assessment

PAH induction was evaluated in the animals. Evaluation criteria included hemodynamic measurements, vascular morphometry, and signs of right‐sided heart failure as observed during sacrifice, namely pleural effusions, ascites, and nutmeg liver due to congestive hepatopathy.

For hemodynamic evaluation, closed‐chest right heart catheterization was performed as described previously [15]. The invasive hemodynamic measurements included mean right atrial pressure (mRAP) and systolic right ventricular pressure (sRVP). Hemodynamic assessment was not possible for two animals of the PAH group because they died during the catheterization. The right ventricular free‐standing wall and the left ventricular free‐standing wall, connected with the intraventricular septum, were weighed for the calculation of Fulton's index. Wet‐to‐dry liver lobe‐weight ratio was calculated to assess congestive hepatopathy.

Four animals did not develop the PAH phenotype. We concluded this based on the sRVP being similar to the controls and the animals lacking signs of right‐sided heart failure pre‐ and post‐termination. Vascular morphometry in those animals showed minimal remodeling. Since the induction of the model had failed, probably due to shunt dysfunction, these four animals were excluded, with one originating from the PAH group and three from the PAH + MCC950 group. In addition, one animal from the PAH + MCC950 group was excluded from further analyses because of extensive adhesions within the abdominal cavity that we identified after sacrifice. Therefore, for the final analyses, there were *n* = 5 animals in the control group, *n* = 9 animals in the PAH group, and *n* = 4 animals in the PAH + MCC950 group.

### 2.4 Human Lung Samples

Human lung tissues (*N* = 14) were collected from patients who had undergone lung transplantation for end‐stage PAH. Transplant patients/caregivers provided written informed consent. This end‐stage PAH cohort included patients with pediatric‐onset PAH associated with congenital heart disease (CHD‐PAH), hereditary PAH (HPAH), or idiopathic PAH (IPAH). These specimens were compared to control lung samples (*N *= 15) obtained from donor lungs, lobectomies, surgical biopsies, or resections. All specimens were free of tumor and fibrosis based on histological evaluation. Two control lung sections exhibited pronounced and widespread medial thickening of the pulmonary arteries and were therefore excluded from subsequent analyses. Characteristics of the end‐stage PAH patients and control patients are shown in Tables 1 and 2.

**Table 1:** Characteristics of pediatric end‐stage PAH patients who underwent lung transplantation.

Case	PAH	Sex	Age at diagnosis	Age at LTx
1	HPAH	F	2.4	17.7
2	HPAH	F	7.0	15.1
3	HPAH	F	15.0	21.6
4	HPAH	F	14.0	16.4
5	HPAH	M	16.3	17.2
6	IPAH	M	8.8	9.5
7	IPAH	M	8.0	9.3
8	IPAH	M	14.9	15.4
9	IPAH	F	7.4	13.8
10	PAH‐CHD	M	13.4	17.7
11	PAH‐CHD	F	5.8	23.8
12	PAH‐CHD	F	14.0	16.9
13	PAH‐CHD	F	12.8	14.3
14	PAH‐CHD	F	12.2	16.2

Abbreviations: HPAH, heritable pulmonary arterial hypertension; IPAH, idiopathic pulmonary arterial hypertension; LTx, lung transplantation; M/F, male/female; PAH–CHD, pulmonary arterial hypertension associated with congenital heart disease.

**Table 2:** Characteristics of control patients from whom lung tissues were obtained.

Case	Age at procedure	Sex	Etiology of lobectomy or bullectomy
1	1	M	Congenital pulmonary adenomatoid malformation
2	18	M	Pulmonary metastasis from osteosarcoma
3	1	M	Congenital pulmonary adenomatoid malformation
4	15	M	Pneumothorax
5	26	M	Non‐seminomatous testicular germ cell tumor metastasis
6	39	F	Metastasis of cervical adenocarcinoma
7	36	F	Metastasis of cervical squamous cell carcinoma
8	0	M	Congenital lobar overinflation
9	1	M	Congenital pulmonary adenomatoid malformation
10	28	M	Synovial sarcoma metastasis
11	1	F	Congenital pulmonary adenomatoid malformation
12	1	F	Congenital pulmonary adenomatoid malformation
13	UNK	UNK	Partially unused allograft due to anatomical incompatibility

Abbreviations: M/F, male/female; UNK, unknown.

### 2.5 Histology Immunostainings on Rat and Human Lungs

#### 2.5.1 Rat Lungs

Formalin‐fixed paraffin‐embedded lungs were cut into 4 µm sections. Pulmonary sections were stained with hematoxylin‐eosin and elastin (HT25A‐1KT; Sigma‐Aldrich) for qualitative and quantitative morphometric analysis of pulmonary arterioles and scanned employing a Hamamatsu NanoZoomer 2.0HT digital slide scanner at 40× magnification (Hamamatsu Photonics). Lung sections were assessed for tissue collapse, defined as tissues exhibiting loss of normal lung architecture. Specimens with over 50% of tissue collapse were excluded from further analysis. In total, three specimens from the PAH group were excluded on this basis. After blinding, each lung tissue scan was divided into four quadrants. In every quadrant, the first five intra‐acinar arterioles (with diameter < 50 µm) that fulfilled the selection criteria as previously described were selected and used for further analysis [16]. External and internal arteriolar areas, medial and neointimal thickness were measured using FIJI‐ImageJ (NIH Image), and the arteriolar occlusion score was calculated as described previously [15]. The percentages of muscularized pulmonary arterioles and neointimal lesions were calculated according to previously published protocols [15]. To assess fibrosis, Sirius Red staining was performed (Direct Red 80, 365548, Sigma Aldrich) (0.1% [w/v]) in 1.3% aqueous picric acid solution (P6744‐1GA, Sigma Aldrich) for 1 h and subsequently two washes in acidified water (1% glacial acetic acid solution; prepared by adding 5 mL of stock solution [Merck 1.00063.1000] to 1 L H_2_O). Collagen content in the vascular wall was assessed in 20 randomly chosen pulmonary arterioles (< 50 μm). Sirius Red positive pixels were isolated from the region of interest with color deconvolution in FIJI. For each arteriole, the percentage of Sirius Red positive pixels relative to the region of interest was calculated, after which 20 measurements were averaged for each animal.

Immunohistochemical staining was performed for CD68 and Ki67. For CD68 staining, antigen retrieval was performed using Tris/EDTA buffer (pH 9). After blocking with normal goat serum (NGS) 10% in PBS, lung sections were incubated with mouse anti‐CD68 primary antibody (MCA341GA, Bio‐Rad [1:100]), and biotinylated horse anti‐mouse IgG secondary antibody (BA‐9500, Vector Laboratories [1:125]). The VECTASTAIN Elite ABC‐HRP complex (PK‐4000, Vector Laboratories) was prepared and applied to sections according to the manufacturer's instructions. Color development was achieved using aminoethyl carbazole chromogen, after which sections were counterstained with hematoxylin. For Ki67 staining, antigen retrieval was performed with citrate (pH 6). Sections were then blocked with NGS 10% diluted in PBS, followed by overnight incubation (4°C) with anti‐Ki‐67 primary antibody (RM‐9106‐s0, Fisher Scientific [1:50]). Next, slides were incubated with biotinylated goat anti‐rabbit secondary antibody (BA‐1000, Vector Laboratories) and conjugated with the ABC‐peroxidase (PK‐4000, Vector). The signal was visualized with 3, 3‐diaminobenzidine (DAB). Sections were counterstained with hematoxylin and mounted with Permount mounting medium (SP15‐100, Fisher Scientific).

Whole slide images were acquired with the Hamamatsu NanoZoomer 2.0HT digital slide scanner (Hamamatsu Photonics) at 40× magnification. Twenty intra‐acinar arterioles (< 50 μm) per lung section were randomly selected. CD68^+^ cells were counted in the vascular wall and perivascular space. The perivascular space was defined as the region within 50 µm from the outer vessel border. Ki67^+^ cells were counted within the vascular wall. Automatic cell quantification was performed with the native segmentation tool of QuPath (v0.6, https://qupath.github.io). The number of positive cells per 10 arterioles was used for statistical analysis.

Immunofluorescent staining was performed for NLRP3 or AIM2, in combination with anti‐CD68. Antigen retrieval was done using citrate (pH 6). Sections were incubated overnight at 4°C with the primary antibodies anti‐NLRP3 (NLRP3/NALP3 antibody [NBP2‐12446], Novus Biologicals [1:100]) or anti‐AIM2 (14‐6008‐93, Polyclonal Antibody, eBioscience [1:100]) on separate sections, each together with anti‐CD68 (MCA341, ED1 clone; Bio‐Rad Laboratories [1:100]). The sections were washed and incubated with the secondary antibodies, goat anti‐rabbit Alexa Fluor 488 (A‐11008, Thermo Fisher Scientific [1:100]) for NLRP3 and AIM2, and goat anti‐mouse Alexa Fluor 647 (Ab150115, Abcam [1:100]) for CD68. Subsequently, sections were counterstained and mounted with ProLong Gold Antifade Mountant with 4′6‐diamidino‐2‐phenylindole (DAPI) (P36941; Invitrogen).

On another set of lung sections, immunofluorescent staining was performed for Ki67 and alpha‐smooth muscle actin (α‐SMA). Sections were stained with the primary antibodies rabbit anti‐Ki67 (RM‐9106‐S0, Thermo Fisher Scientific [1:50]) and mouse anti‐α‐SMA (67735‐1‐Ig; Proteintech [1:100]). Next, sections were incubated with the secondary antibodies goat anti‐rabbit Alexa Fluor 488 (A‐11034; Thermo Fisher Scientific [1:200]) and goat anti‐mouse Alexa Fluor 647 (ab150115; Abcam [1:200]). Subsequently, sections were mounted with ProLong Gold Antifade Mountant DAPI (P36941; Invitrogen). An overview of the antibodies used for immunostainings is provided in Supporting Information S1: Table 1.

#### 2.5.2 Human Lungs

Formalin‐fixed paraffin‐embedded lungs were cut into 4 µm sections. Pulmonary sections were stained with hematoxylin‐eosin and elastin (HT25A‐1KT; Sigma‐Aldrich) for qualitative and quantitative morphometric analysis of pulmonary arterioles and scanned employing a Hamamatsu NanoZoomer 2.0HT digital slide scanner at 40× magnification (Hamamatsu Photonics). Subsequently, immunofluorescent staining was performed on lung sections for NLRP3 or AIM2, each in combination with anti‐CD68. Antigen retrieval and immunostaining were performed as described above. Lung sections were incubated with the same antibodies against NLRP3 (1:50) and AIM2 (1:250) as used for rat lung tissue. For CD68 detection in human lung tissue, anti‐CD68 (MA5‐13324; Thermo Fisher Scientific [1:100]) was used. Secondary antibodies included goat anti‐rabbit Alexa‐Fluor 488 (A‐11034, Thermo Fisher Scientific [1:100]) and goat anti‐mouse Alexa Fluor 647 (Ab150115, Abcam). Subsequently, sections were mounted with ProLong Gold Antifade Mountant DAPI (P36941; Invitrogen).

Immunofluorescent images were captured with the Zeiss 410 inverted laser scan microscope (Leica Microsystems), ApoTome.2 (Zeiss) and processed with Zeiss Zen (Carl Zeiss Microscopy GmbH) and Qupath (v0.6; https://qupath.github.io). Ki67‐α‐SMA positive cells were quantified within the vascular wall of 20 randomly chosen pulmonary intra‐acinar arterioles per section.

### 2.6 Immunoblotting

Rat lung tissues were snap‐frozen, and protein was isolated. For caspase‐1, IL‐1β, IL‐18, and AIM2 immunoblotting, wet membrane transfer was performed. Anti‐caspase‐1 (14F468) (1:200) (sc‐56036; Santa Cruz Biotechnology Inc.), anti‐IL‐1β (1:1000) (IL‐1β IL‐F2 NBP1‐42767; Novus Biologicals), anti‐IL‐18 (1 µg/mL) (IL‐18/IL‐1F4, AF521; R&D Systems) and anti‐AIM2 (1:1000) (AIM2 Polyclonal Antibody, eBioscience 14‐6008‐93, eBioscience, Inc.) and anti‐GAPDH (1:10000) (ab8245, Abcam plc) were used as primary antibodies and anti‐rabbit IgG HRP (1:2000) (#1706515; Bio‐Rad Laboratories Inc.) or anti‐goat IgG HRP‐linked antibody (1:2000) (sc‐2020; Santa Cruz Biotechnology Inc.) as secondary antibodies, respectively. GAPDH was used as loading control. All antibodies were diluted in 2.5% fat‐free milk in TBS‐T. FIJI‐ImageJ (NIH) was used for the densitometry of the immunoblots.

### 2.7 Statistical Analysis

After exclusion of rats due to failure to induce the PAH phenotype in four rats and one rat showing extensive adhesions within the abdominal cavity that we identified after sacrifice, there were, as stated above, *n* = 5 animals in the control group, *n* = 9 animals in the PAH group and *n* = 4 animals in the PAH + MCC950 group. Since there were *n* < 6 for animals in multiple groups, all variables were assessed with non‐parametric tests. For the natural history study that included *n* = 5 rats per group, multiple group comparisons were evaluated with the Kruskal‐Wallis test followed by Dunn's post hoc test. Differences between the control and PAH or PAH and PAH + MCC950 groups were assessed using one‐tailed Mann–Whitney *U* test. *p* ≤ 0.05 was considered statistically significant. All statistical analyses were performed using GraphPad Prism 10 (GraphPad Software). Results are shown as means ± standard error of the mean (SEM).

## 3 Results

### 3.1 Inflammasomes Are Gradually Activated During PAH

**Figure 1:**
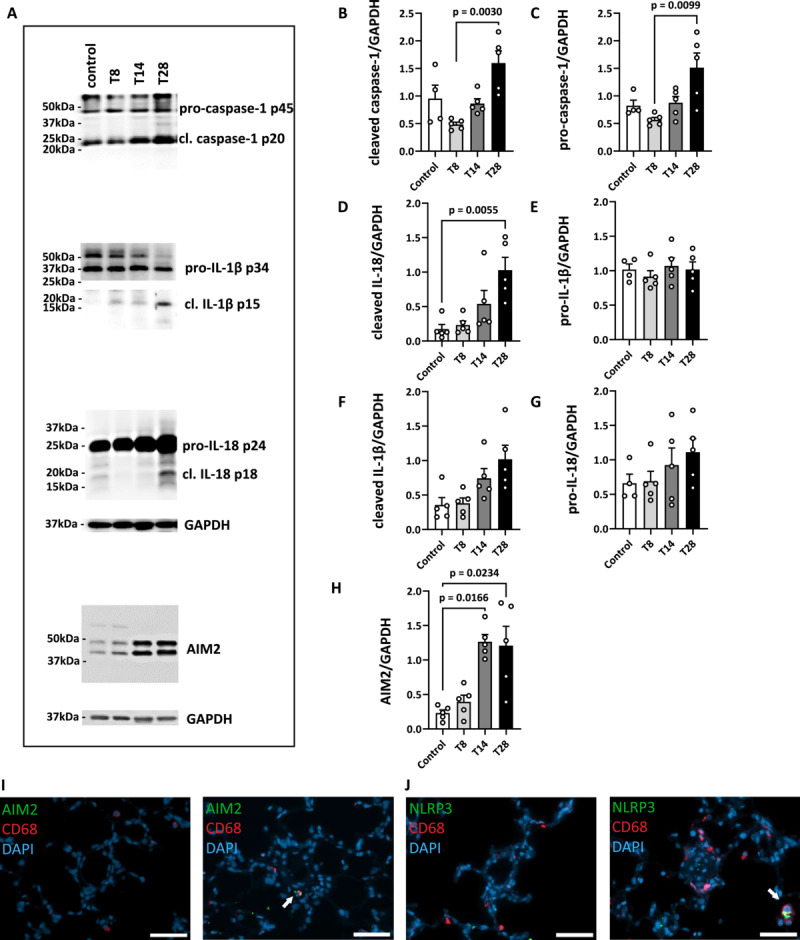
Natural history of inflammasome activation in lungs of aortocaval shunt and monocrotaline rat model of PAH. PAH was induced in rats by injection of monocrotaline (60 mg/kg in NaCl 0.9%, subcutaneous injection) followed by aortocaval shunt surgery 7 days later. Controls received injections with NaCl 0.9% and underwent sham surgery. Lungs were collected 1 (*T* = 8), 8 (*T* = 14), or 21 days (*T* = 28) after aortocaval shunt surgery, or *T* = 28 after sham surgery (controls). Lung tissue was homogenized and caspase‐1, interleukin (IL)‐1β, IL‐18, and AIM2 were assessed by Western blot. (A) Representative immunoblots; (B–H) Quantification of cleaved caspase‐1, pro‐caspase‐1, cleaved IL‐1β, pro‐IL‐1β, cleaved IL‐18, pro‐IL‐18, and AIM2 using GAPDH as loading control. (I, J) Representative immunofluorescence images of pulmonary arterioles in control and PAH lung sections. (I) Co‐localization of perivascular NLRP3 (green) and macrophage marker CD68 (red). (J) Perivascular colocalization of AIM2 and CD68. Scale bars correspond to 50 μm. Data are shown as mean ± SEM. Kruskal–Wallis test with Dunn's multiple comparisons test was used to detect differences between groups. Exact *p* values (where *p* < 0.05) are indicated on the graphs. *n* = 5 rats per group. PAH, pulmonary arterial hypertension.

**Figure 2:**
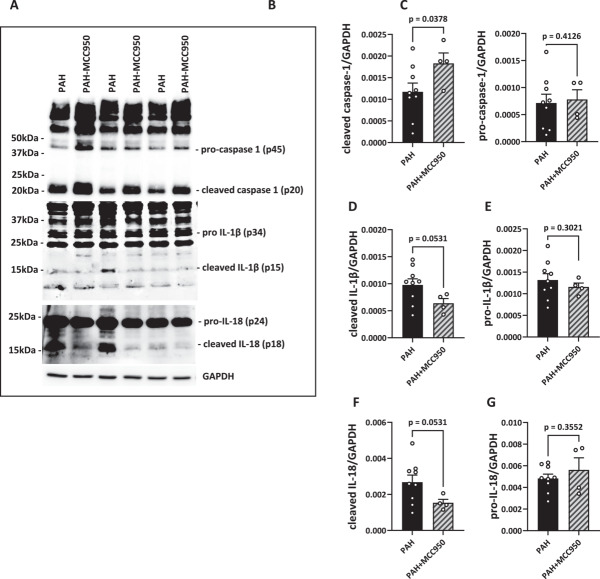
Effect of MCC950 on inflammasome activation in the lungs of the aortocaval shunt and monocrotaline rat model of PAH. After aortocaval shunt surgery and monocrotaline injection (as described in the legends of Figure 1), PAH rats received MCC950 injections (10 mg/kg in PBS daily, intraperitoneal) or vehicle (PBS injections, intraperitoneal) from the day after surgery onwards. On Day 28, animals were sacrificed by exsanguination from the abdominal aorta, and lungs were collected. Lung tissue was homogenized, and caspase‐1, IL‐1β, and IL‐18 were assessed by Western blot. (A) Representative immunoblots. (B–G) Quantification of cleaved caspase‐1, pro‐caspase‐1, cleaved IL‐1β, pro‐IL‐1β, cleaved IL‐18, and pro‐IL‐18. Data are shown as mean ± SEM. The differences between the PAH and the PAH + MCC950 group were assessed using one‐tailed Mann–Whitney test. Exact *p* values are indicated on the graphs. PAH, pulmonary arterial hypertension.

**Figure 3:**
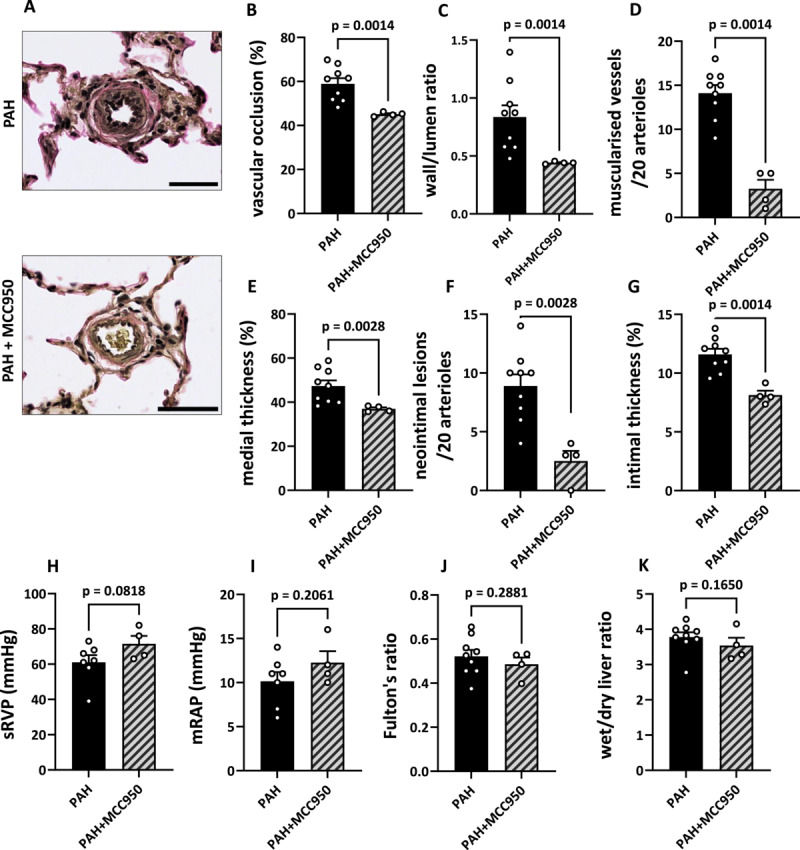
Effect of MCC950 on pulmonary vascular remodeling and hemodynamics in the aortocaval shunt and monocrotaline rat model of PAH. Lungs collected 21 days after aortocaval shunt surgery were embedded in paraffin, sectioned (4 µm), and stained for Verhoeff's elastin. 20 intra‐acinar pulmonary arterioles per animal were randomly selected for quantitative morphometric analyses. (A) Representative images of pulmonary intra‐acinar arterioles in PAH and PAH + MCC950 rats. Scale bar: 50 μm. (B–G) Wall to lumen ratio, quantification of vascular occlusion, proportion of muscularised vessels, medial thickness, proportion of neointimal lesions, and intimal thickness. (H, I) Hemodynamic parameters were assessed 21 days after aortocaval shunt surgery. Hemodynamic assessment was not possible for two animals of the PAH group because they died during the catheterization. Quantification of hemodynamic parameters included right ventricular systolic pressure (sRVP), mean right atrial pressure (mRAP), (J) Fulton's ratio, and (K) liver's wet to dry weight ratio. Data are shown as mean ± SEM. The differences between the PAH and the PAH + MCC950 group were assessed using one‐tailed Mann–Whitney test. Exact *p* values are indicated on the graphs.

**Figure 4:**
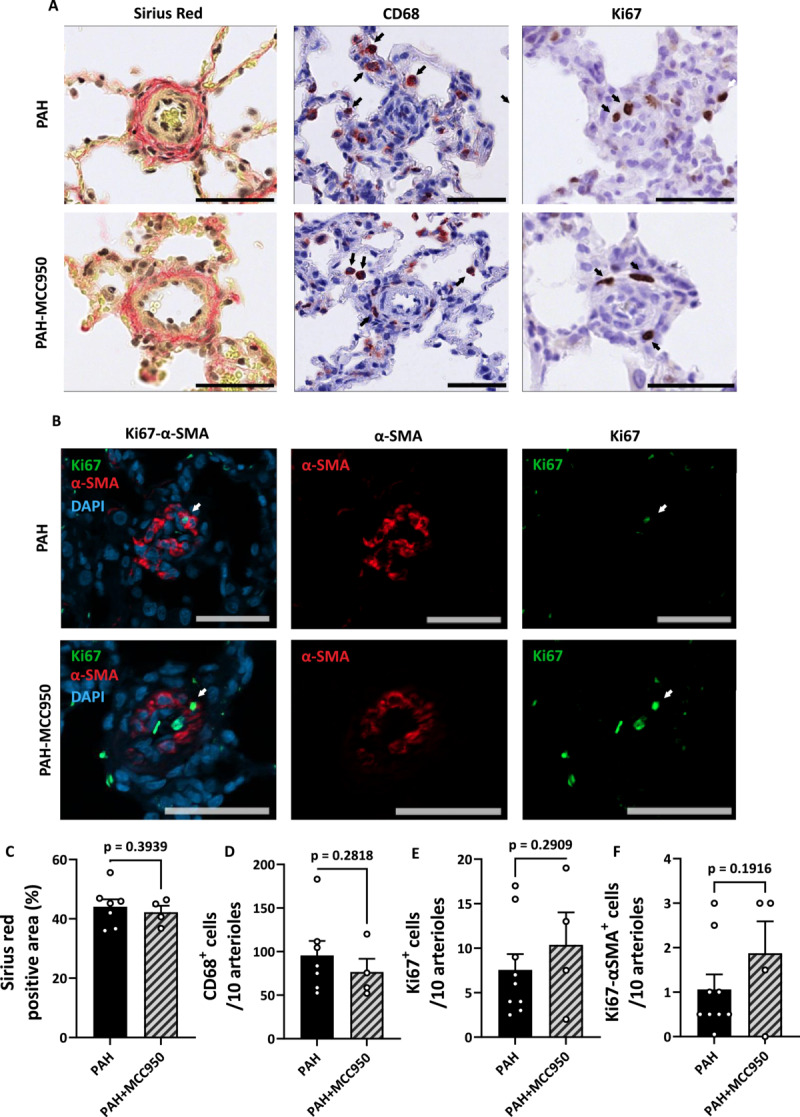
Effect of MCC950 on collagen deposition, macrophage infiltration, and cell proliferation. (A) Representative images for Sirius Red (collagen staining), CD68 (macrophage marker), Ki67 (proliferation marker), and dual immunofluorescence (IF) staining for Ki67 and ⍺‐SMA (smooth muscle cell marker), in vehicle, and MCC950 treated PAH groups, with CD68^+^ perivascular and transmural cells indicated by black arrows (middle, left), Ki67^+^ cells in the vascular wall of intra‐acinar vessels indicated by black arrows (middle, right), and (B) the overlay between Ki67^+^ cells (green) and ⍺‐SMA (red) indicated by white arrows (right); (C) Sirius Red staining was quantified as mean collagen positive area from 20 randomly selected pulmonary intra‐acinar vessels (< 50 µm diameter) per rat. (D) CD68 was quantified as the mean number of positive cells in 20 vessels per rat. Cells were counted within a ROI 50 μm from the outer vessel border. (E) Ki67^+^ cells were quantified as the mean number of positive cells in 20 vessels per rat. (F) Overlay of Ki67^+^ and ⍺‐SMA^+^ cells within the vascular wall for PAH and PAH + MCC950 groups. Two animals were excluded from the PAH group due to collapsed lung tissue. Data are shown as mean ± SEM and were analyzed by one‐tailed Mann–Whitney test. Scale bar: 50 μm. Exact *p* values are indicated on the graphs.

We investigated inflammasome activation in the MCT/ACS rat model of neointimal PAH on lung tissue, where PAH animals had been sacrificed on Day 8 (T8), reflecting the acute response to hemodynamic loading on injured endothelium, Day 14 (T14) to study early‐stage flow‐associated PAH, and Day 28 (T28) for late‐stage neointimal disease. We observed a trend towards a progressive increase in cleaved caspase‐1, IL‐1β and IL‐18 over time that reached statistical significance at T28 compared to T8 for cleaved caspase‐1, and at T28 compared to controls for cleaved IL‐18 (Figure 1A,B,D,F), while the pro‐form of caspase‐1 also was increased at T28 compared to T8 (Figure 1C), but the pro‐forms of IL‐1β and IL‐18 were unchanged (Figure 1E,G). We then assessed the presence of the AIM2 inflammasome employing Western blot and found increased AIM2 at T14 and T28 compared to controls (Figure 1A,H). Whole uncut blots are shown in Supporting Information S1: Figures 1–4. This was complemented by the presence of AIM2^+^ cells and AIM2^+^CD68^+^ macrophages in PAH rat lung tissue as assessed by immunofluorescent stainings at T28 (Figure 1I). Similar to our previous studies [6], we found NLRP3^+^CD68^+^ macrophages in PAH lung tissue at T28 (Figure 1J). Together, these findings show that inflammasome activation increases over time in the MCT/ACS rat model of neointimal PAH, while reaching statistical significance for cleaved caspase‐1 and IL‐18 only at T28, which reflects end‐stage PAH. The immunofluorescent data show the presence of the NLRP3 and AIM2 inflammasomes, suggesting upregulation of both these inflammasomes in the MCT/ACS rat model of neointimal PAH.

### 3.2 MCC950 Tends to Suppress NLRP3 Inflammasome Activation in the MCT/ACS Rat Model of Neointimal PAH

**Figure 5:**
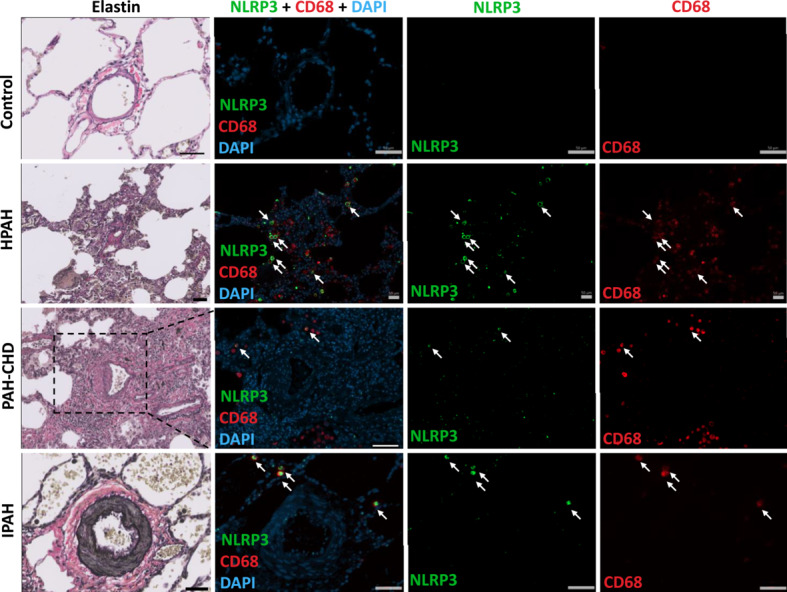
NLRP3 colocalises with CD68 in end‐stage PAH lung sections. Lung tissue samples were obtained from control subjects without pulmonary vascular disease and from patients with heritable pulmonary arterial hypertension (HPAH), idiopathic PAH (IPAH), and congenital heart disease‐associated PAH (CHD‐PAH). Lung sections were stained for Verhoeff's elastin. Adjacent sections were stained for NLRP3 and CD68. Panel shows representative images of pulmonary arteries stained with Verhoeff's elastin and the corresponding vessels stained for NLRP3. White arrows point to NLRP3^+^‐CD68^+^ cells. Scale bars correspond to 50 μm.

**Figure 6:**
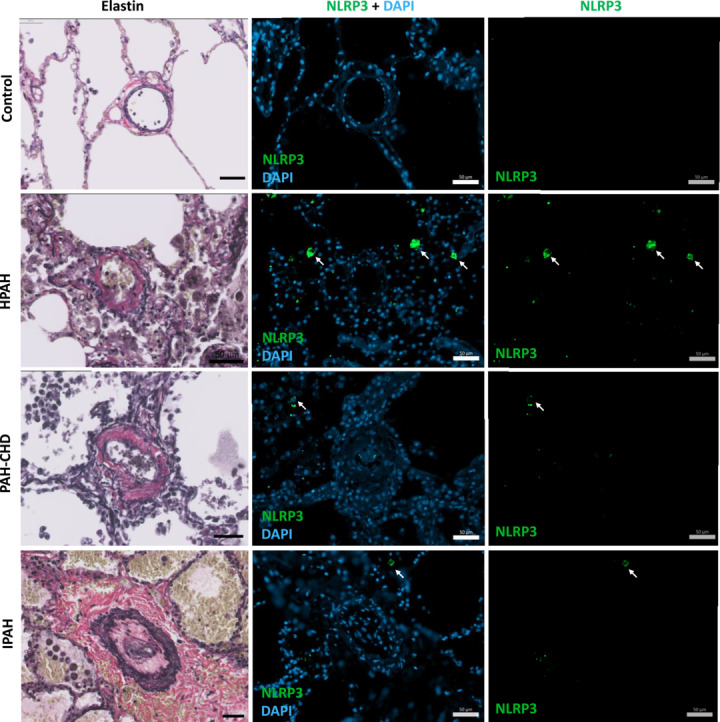
NLRP3 expression in explanted lung tissue from end‐stage PAH patients. Lung tissues were obtained from control subjects without pulmonary vascular disease and from patients with heritable pulmonary arterial hypertension (HPAH), idiopathic PAH (IPAH), and congenital heart disease‐associated PAH (CHD‐PAH). Lung sections were stained for Verhoeff's elastin. Adjacent sections were stained for NLRP3. Panel shows representative images of pulmonary arteries stained with Verhoeff's elastin and the corresponding vessels stained for NLRP3. White arrows point to NLRP3^+^ cells. Scale bars correspond to 50 μm.

**Figure 7:**
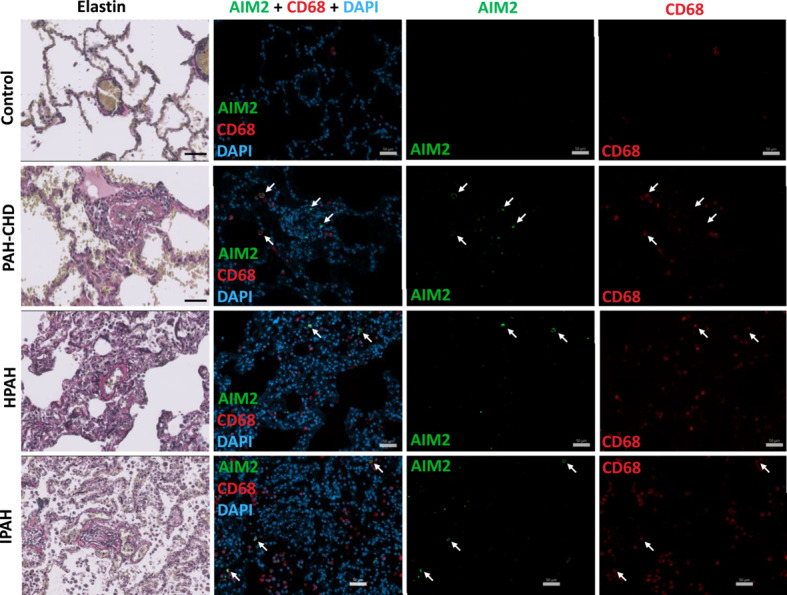
AIM2 Expression in Explanted Lung tissue from End‐stage PAH patients. Lung tissues were obtained from control subjects without pulmonary vascular disease and from patients with heritable pulmonary arterial hypertension (HPAH), idiopathic PAH (IPAH), and congenital heart disease‐associated PAH (CHD‐PAH). Lung sections were stained for Verhoeff's elastin. Adjacent sections were stained for AIM2. Panel shows representative images of pulmonary arteries stained with Verhoeff's elastin and the corresponding vessels stained for AIM2. White arrows point to AIM2^+^ cells. Scale bars correspond to 50 μm.

We then investigated whether NLRP3 inflammasome inhibition by MCC950 may ameliorate PAH. We injected MCC950 dissolved in PBS (PAH + MCC950 group) or PBS only (PAH group) into PAH rats on a daily basis starting on the day after aortocaval shunt surgery (Day 8) until termination (Day 28). After we sacrificed the rats, we examined inflammasome activation in lung homogenates. While MCC950 unexpectedly increased cleaved caspase‐1 (Figure 2A,B), the increase in cleaved caspase‐1 was not reflected by increases in the cleavage of IL‐1β and IL‐18. As expected, cleaved IL‐1β and IL‐18 showed a strong trend towards a decrease that almost reached statistical significance (*p *= 0.0531; Figure 2A,B,D,F). The proforms of caspase‐1, IL‐1β, and IL‐18, were, as expected, unchanged (Figure 2A,C,E,G). Whole uncut blots are shown in Supporting Information S1: Figures 5–8. AIM2 protein levels did not show a difference between PAH and PAH + MCC950 (Supporting Information S1: Figure 9).

### 3.3 MCC950 Attenuates Pulmonary Vascular Remodeling, but Does Not Improve Pulmonary Hemodynamics in the MCT/ACS Rat Model of Neointimal PAH

Similar to previous observations [15], the MCT/ACS rat model of neointimal PAH displayed advanced vascular remodeling compared to the control animals. This included muscularization of the pulmonary arterioles and neointima formation, quantified as medial and intimal thickness, which cumulatively counts for vascular occlusion (Supporting Information S1: Figure 10). Treatment with MCC950 reduced vascular remodeling. This was reflected by a 24% (*p *= 0.001) decrease in vascular occlusion compared to the untreated PAH group (Figure 3A,B), and effects on the media and intima of lung arterioles (Figure 3C). The count of muscularized arterioles was reduced by 77% (*p *= 0.001) and this was also reflected in decreased medial thickness (22%, *p*= 0.003) in the MCC950 group compared to the untreated group (Figure 3D,E). The neointima formation was reduced by 70% (*p *= 0.003) as reflected by a 33% (*p *= 0.001) decrease in intimal thickness in animals treated with MCC950 compared to the untreated group (Figure 3F,G). Treatment with MCC950 did not affect the hemodynamic condition of the animals, as assessed by sRVP and mRAP (Figure 3H,I). Similarly, the Fulton's index, reflecting right ventricular hypertrophy, and the liver's wet to dry weight ratio revealed no differences between the groups (Figure 3J,K).

The inflammasome end‐product IL‐1β promotes the recruitment of macrophages, proliferation of vascular smooth muscle cells, and fibrosis [9, 10, 16]. Even though the intra‐acinar pulmonary arterioles in the animals that received MCC950 treatment demonstrated decreased remodeling compared to the untreated group, fibrosis, as assessed by Sirius Red staining, was not affected (Figure 4A–C). The number of CD68^+^ macrophages in the vessel wall and the perivascular space was not affected by treatment with MCC950 (Figure 4A,D). The cell proliferation, characteristic of vascular remodeling, was assessed in the total wall and specifically in the α‐SMA^+^Ki67^+^ cells. MCC950 did not affect total Ki67 staining or α‐SMA^+^ Ki67^+^ cells, consistent with no effects on cellular proliferation (Figure 4A,E,F). The comparison between the control animals and the PAH group is provided in Supporting Information S1: Figure 11.

### 3.4 NLRP3 and AIM2 Are Expressed in Pediatric End‐Stage PAH Lungs

To assess the translational relevance of our data, we then examined NLRP3 and AIM2 in lung samples of 14 pediatric patients with end‐stage PAH and 13 controls (Tables 1 and 2).

Immunofluorescent staining of NLRP3 demonstrated an increase in NLRP3 staining in PAH lungs compared to controls (Figure 5). The NLRP3 expression was variable and did not show a consistent increase in all PAH patient samples that we examined (Figure 5). NLRP3 immunoreactivity was mainly seen in the perivascular space, although some signal was also observed in cells lining the vascular lumen, perhaps suggesting endothelial cell origin (Figure 5). To further characterize the cell type expressing NLRP3, we co‐stained tissues for CD68. NLRP3 staining predominantly co‐localized with CD68 staining in the perivascular space (Figure 5), suggesting, similar to our findings in the PAH rat model, the presence of the NLRP3 inflammasome in pulmonary macrophages. We observed NLRP3^+^ cells in pediatric lungs with CHD‐PAH, IPAH, and HPAH, suggesting that the specific PAH phenotype did not affect the presence of NLRP3^+^ cells, although our sample size may be too small for a definite conclusion (Figure 6).

Next, we evaluated AIM2 in lung tissues. Similar to NLRP3, we observed AIM2 positive staining in lungs from PAH patients irrespective of the specific PAH phenotype (Figure 7). While AIM2 primarily co‐localized with CD68, suggesting the presence of AIM2 in pulmonary macrophages, we also observed AIM2 immunoreactivity in cells not expressing CD68, indicating that, in addition to macrophages, other pulmonary cells may express the AIM2 inflammasome (Figure 7).

## 4 Discussion

Several studies [9, 12], including our own [6], have shown that the NLRP3 inflammasome is activated in PAH. While studies from Al‐Qazazi et al. mainly showed NLRP3 inflammasome activation in right ventricular macrophages, both in the MCT and Sugen hypoxia rat models of PAH, as well as in adult PAH patients [12], we previously found NLRP3 inflammasome activation in pulmonary macrophages and endothelial cells of the MCT/ACS rat model of neointimal PAH in advanced disease [6]. We here show a tendency towards gradual inflammasome activation in pulmonary cells in a natural history study in the MCT/ACS rat model of neointimal PAH, and find that not only expression of the NLRP3, but also the AIM2 inflammasome increases during the development of PAH. Of translational relevance, we found NLRP3 and AIM2 staining on sections of lungs from several, though not all, pediatric end‐stage PAH disease patients, while NLRP3 and AIM2 were undetectable in lungs of patients who we selected as controls (donor lungs from lobectomies, surgical biopsies, or resections). Our studies thus show gradual inflammasome activation during the development of PAH in lungs of the MCT/ACS rat model, and NLRP3 and AIM2 inflammasome expression in pulmonary tissue of end‐stage pediatric PAH patients. These studies may imply a role for inflammasome activation in pulmonary tissue in PAH.

The NLRP3 inflammasome is a therapeutic target in several disease phenotypes, with a large number of drugs currently in clinical trials [17]. The NLRP3 inflammasome inhibitor MCC950 is widely used in preclinical inflammasome studies [18]. While previous studies have shown that MCC950 improved some aspects (tricuspid annular plane systolic excursion, cardiac index) of right ventricular function in the MCT and Sugen hypoxia rat models of PAH [12], we here complement these findings by showing, in the MCT/ACS model of neointimal PAH, that MCC950 reduced pulmonary vascular remodeling, reflected by a decrease in total occlusion, muscularized arterioles, medial thickness, and neointimal formation. The changes in these morphological parameters were not accompanied by effects on fibrosis as assessed by Sirius Red collagen staining, accumulation of CD68^+^ macrophages, or effects on SMC proliferation, leaving the exact mechanism as to how MCC950 reduced pulmonary vascular remodeling insufficiently explained. We did, however, observe that MCC950 decreased active IL‐1β and IL‐18 in rat lung tissue, only just failing to reach statistical significance in this small sample study, suggesting that the decrease in pulmonary vascular remodeling likely occurred downstream of these two interleukins. The decreases in these two interleukins being subtle, probably explained why fibrosis, pulmonary macrophage accumulation, or SMC proliferation were not affected.

Treatment with MCC950 did not affect the hemodynamic condition of the animals, as assessed by sRVP and mRAP, or Fulton's index, reflecting right ventricular hypertrophy, which also may be due to the relatively subtle effect of MCC950 on active IL‐1β and IL‐18 in pulmonary tissue. In seeming contrast, Al‐Qazazi et al. did report hemodynamic improvement upon MCC950 treatment in MCT‐only induced PAH [12]. However, there are several complications in the direct comparison of the results from Al‐Qazazi's study with our study. The model of PAH in Al‐Qazazi's study is less advanced than in ours, which may explain why Al‐Qazazi observed hemodynamic improvement upon MCC950 treatment and we did not. Further, the interpretation of MCC950 not changing sRVP in our model is complicated by potential changes in pulmonary flow due to the shunt in our model. This shunt might mask changes in pulmonary vascular resistance associated with reduced pulmonary vascular remodeling, and therefore, we may not have observed an effect of MCC950 on sRVP. Nevertheless, our data suggest that inflammasome inhibition in itself is unable to ameliorate the hemodynamics in neointimal PAH, induced by the double hit MCT and ACS, and resembling pediatric PAH, whereas in early PAH, characterized by only medial hypertrophy, MCC950 might improve hemodynamics [19, 20]. Alternatively, the difference between the outcome on hemodynamics in the two studies could be due to different dosing of MCC950 and the administration route, where Al‐Qazazi et al. administered MCC950 i.v. at a lower dose (6 mg/kg) [12], and we used i.p. treatment of 10 mg/kg MCC950, a dose used in previous studies employing long‐term treatment of MCC950 in rats [21].

Of note, in a prior study where we investigated the role of erythropoietin in the interplay between endothelial progenitor cells and heme oxygenase‐1, we also found no effects on hemodynamic parameters in the MCT/ACS model of PAH, while pulmonary vascular remodeling was decreased [22]. These findings indicate that reduced pulmonary vascular remodeling can occur in the absence of effects on hemodynamic parameters. Thus, we here elucidate a main role for the NLRP3 inflammasome in pulmonary vascular remodeling, an aspect of PAH that can be ameliorated by NLRP3 inflammasome inhibition.

Our study has several limitations, including the relatively small number of animals in experimental groups. In the natural history study, we used, by taking into account the reduction of animals in research, samples from an earlier study, with *n* = 5. For this reason, we may not have seen significant differences in inflammasome activation parameters at early timepoints, while inflammasome activation did increase over time, reaching significance at Day 28, similar to our previous observations [6]. In addition, unfortunately, a large number of animals included in the PAH + MCC950 group did not develop PAH (*n* = 3), while one animal had to be excluded because of extensive adhesions within the abdominal cavity that we identified after sacrifice. We therefore only had *n* = 4 animals in the PAH + MCC950 group. This may have contributed to the subtle differences of MCC950 on active IL‐1β and IL‐18, while we did observe clear differences in vascular remodeling. In addition, while we managed to include lung tissues from 14 pediatric PAH patients with different PAH phenotypes for immunostainings, this number did not allow us to detect differences in NLRP3 and AIM2 stainings between different PAH phenotypes, leaving us with the more general conclusion that NLRP3 and AIM2 are expressed at the protein level in pediatric PAH lungs.

Together, we here provide evidence for inflammasome activation in pediatric PAH and show that NLRP3 inflammasome inhibition ameliorates vascular remodeling in the MCT/ACS model of neointimal PAH. The latter suggests that the NLRP3 inflammasome inhibitors currently being tested in clinical trials for coronary heart disease, cryopyrin periodic syndromes, and Parkinson's disease [17] may have a broader therapeutic applicability.

## Author Contributions

Emmanouil Mavrogiannis, Rebeca Weldeghebreal, and Iris R. Schilthuis participated in study design, data acquisition, data interpretation, statistical analysis, and drafting of the manuscript. Zain K. Fal participated in data acquisition, data interpretation, and statistical analysis. Niels J. Kloosterhuis and Mirjam H. Koster participated in data acquisition and data interpretation. Wim Timens participated in data acquisition and data interpretation. Johannes M. Douwes, Rolf M. F. Berger, and Marit Westerterp participated in study design, data interpretation, statistical analysis, drafting, and revising of the manuscript. All authors revised the manuscript critically for important intellectual content and approved the manuscript submission.

## Ethics Statement

License 6911A, 2316713‐1‐1 (animal studies); METc 2008.009 (human studies).

## Conflicts of Interest

The University Medical Center Groningen (UMCG) contracts with Johnson&Johnson/Actelion, MSD, and Liquidia for steering committee and advisory board activities of Rolf M. F. Berger outside the content of this manuscript. The other authors declare no conflicts of interest.

## Supporting information

Supporting File

## Data Availability

The data that support the findings of this study are available from the corresponding author upon reasonable request.
