# Supplementary material for: The NLRP3 Inflammasome Increases Pulmonary Vascular Remodeling in Experimental Pulmonary Arterial Hypertension
Source: Pulm Circ. 2026 Jul 17;16(3):e70354. doi: 10.1002/pul2.70354 (PMC13404779; doi:10.1002/pul2.70354)
Supplement: Supplementary file 1 — Supporting File [file PUL2-16-e70354-s001.pdf]

Supplemental Figure 1

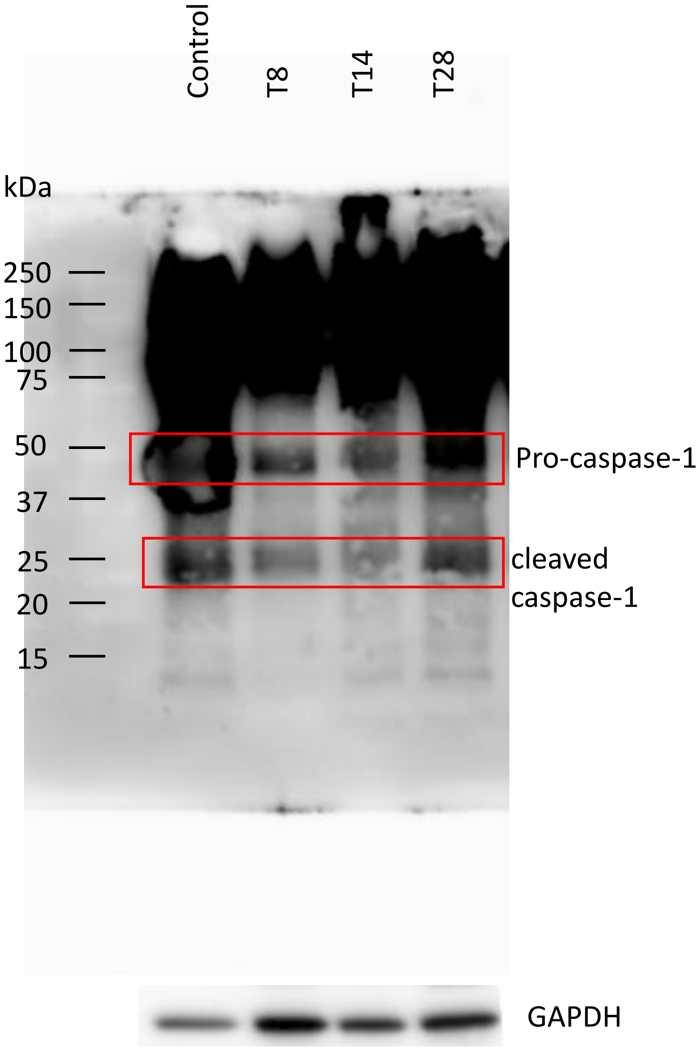

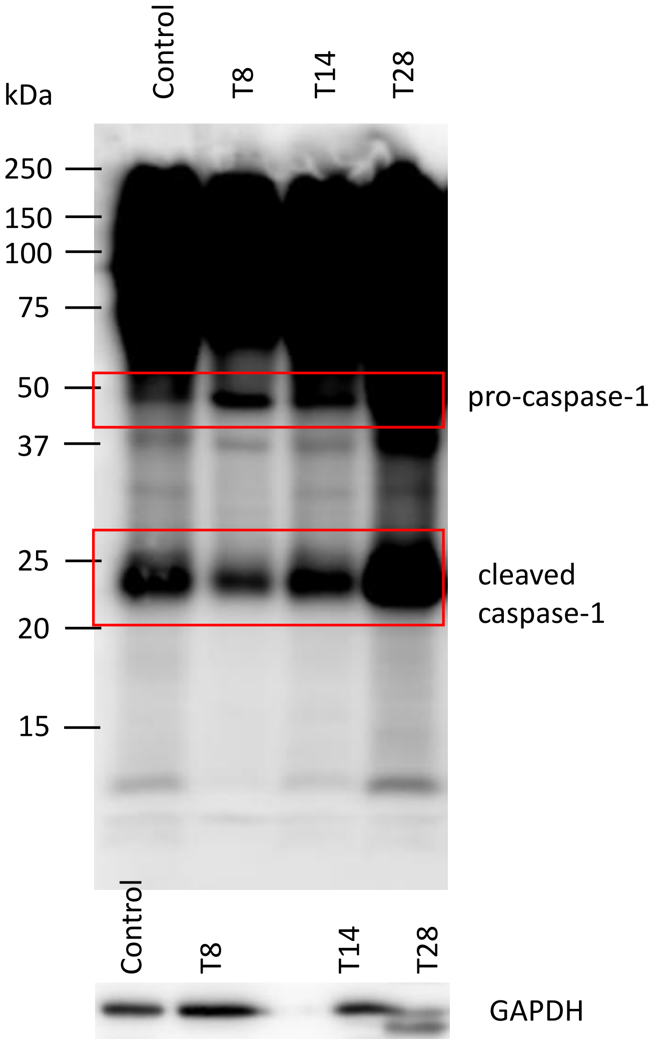

Supplemental Figure 1 (continued)

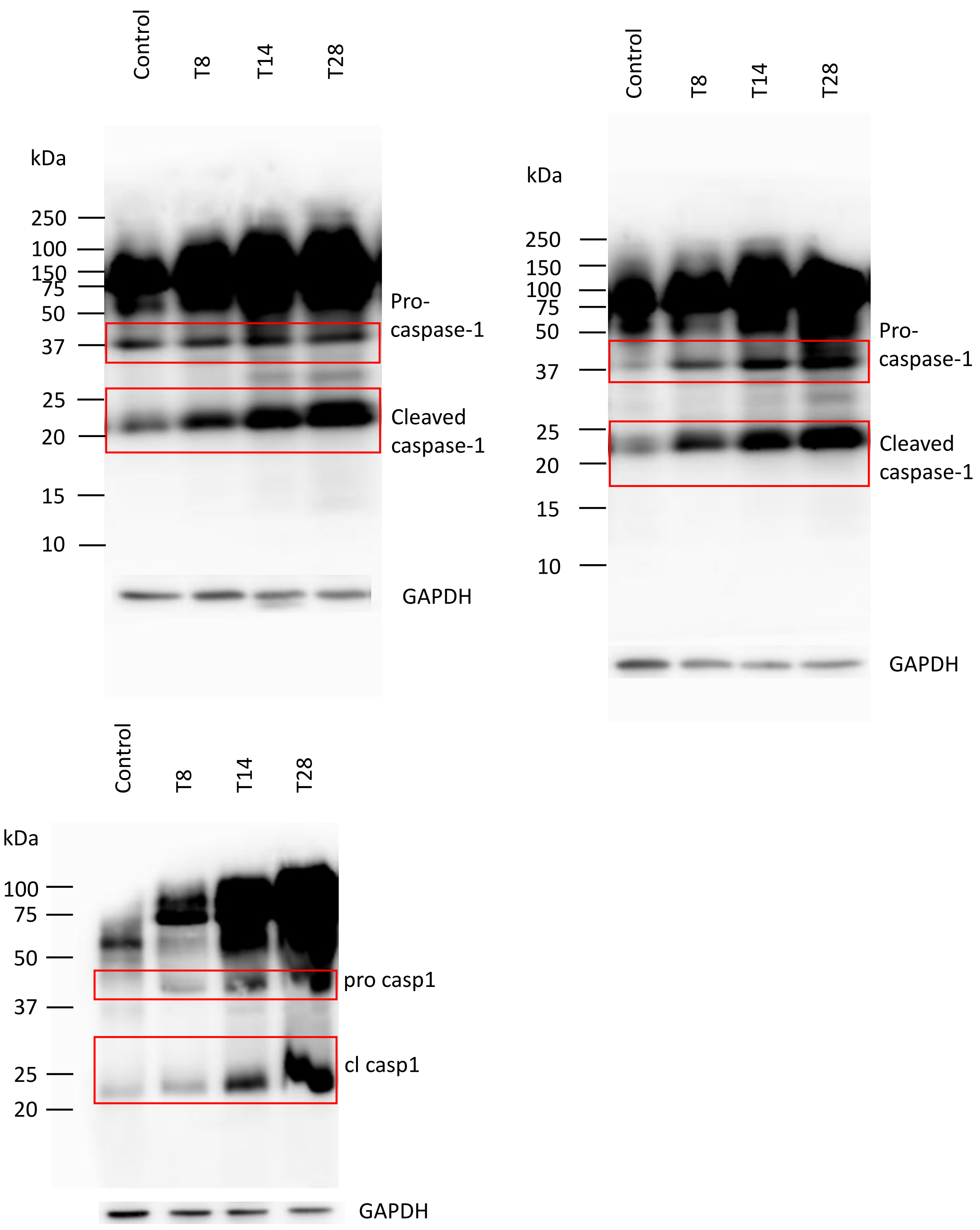

Supplemental Figure 1. Unedited Western blots of caspase-1, used for quantification in figure 1. Parts of the Western blots used for quantification are boxed.

Supplemental Figure 2

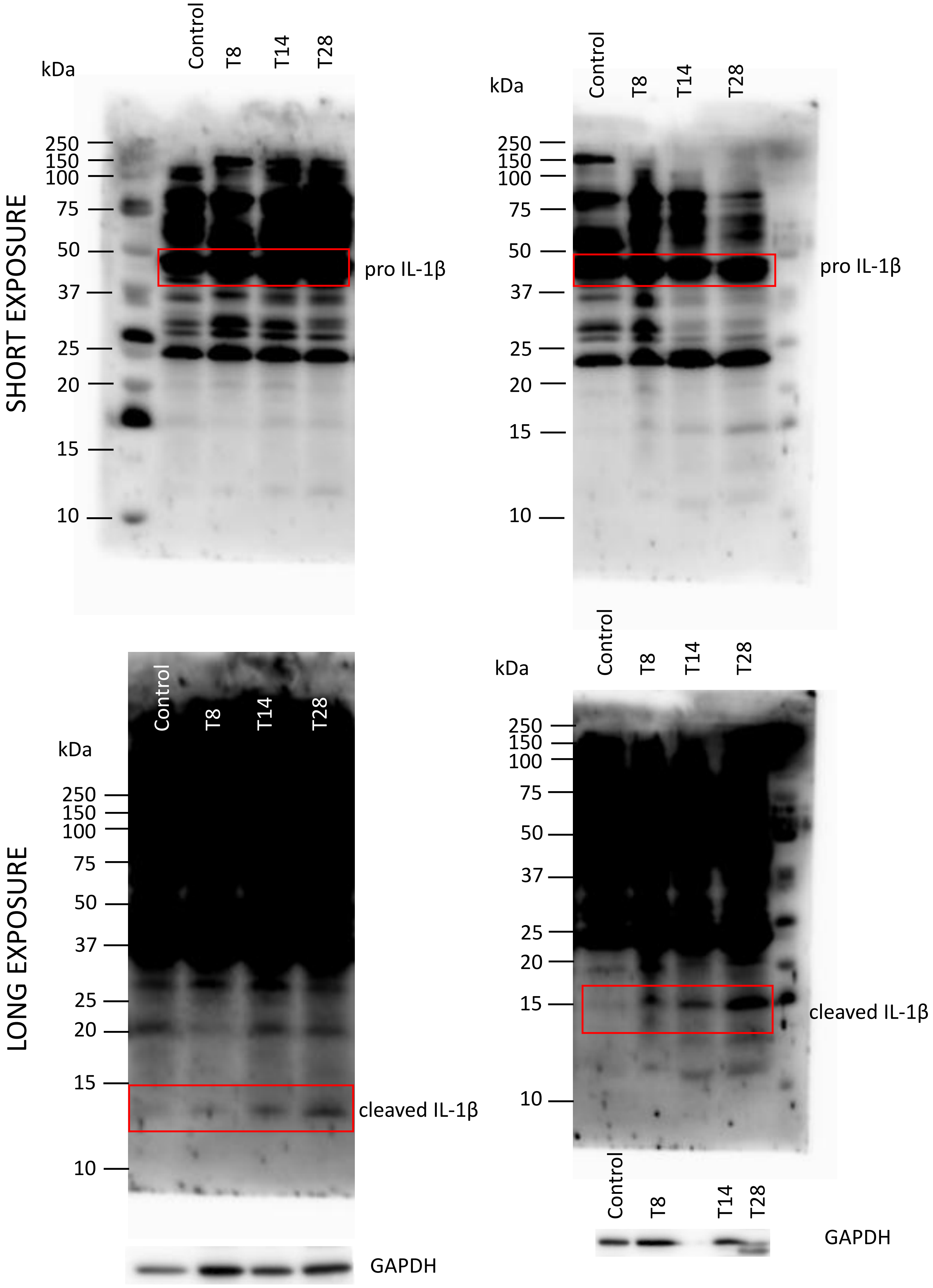

Supplemental Figure 2 (continued)

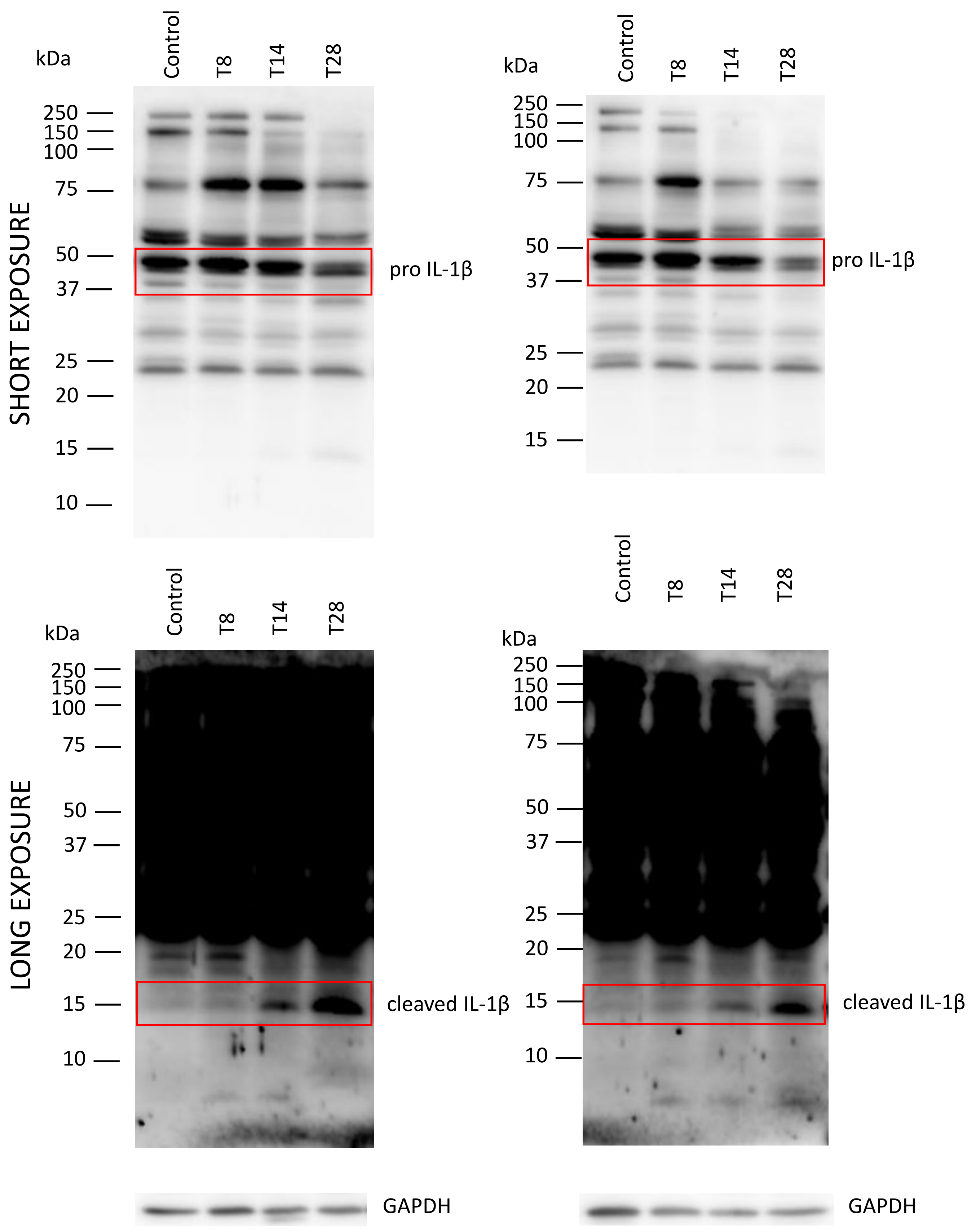

Supplemental Figure 2 (continued)

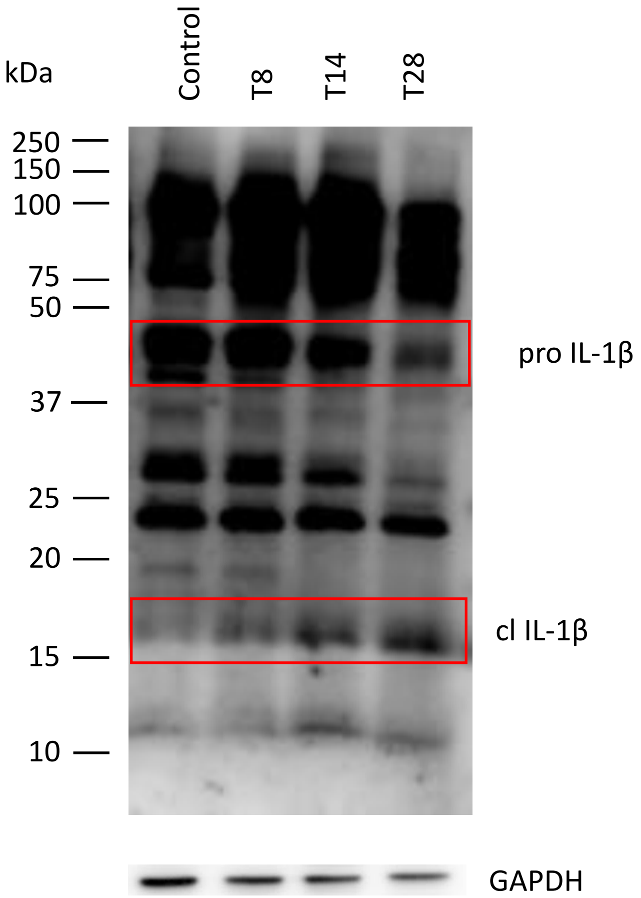

Supplemental Figure 2. Unedited Western blots of IL-1β, used for quantification in figure 1. Parts of the Western blots used for quantification are boxed. Short and long exposure of the same blot is provided for better visualization of the pro-IL-1β, and cleaved IL-1β, respectively.

Supplemental Figure 3

A

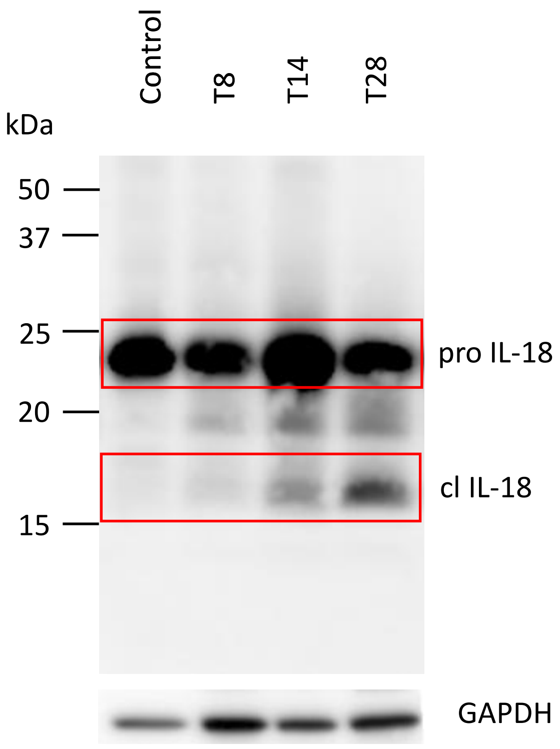

B

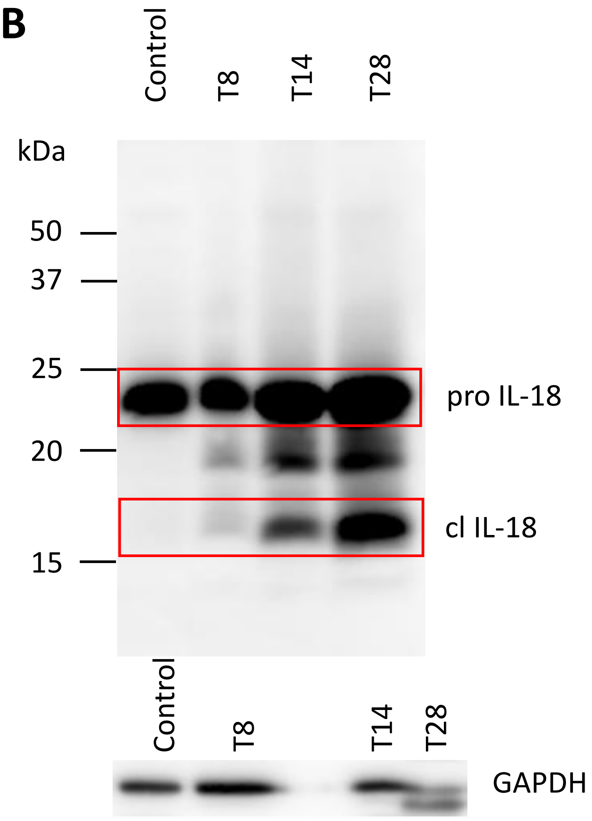

C

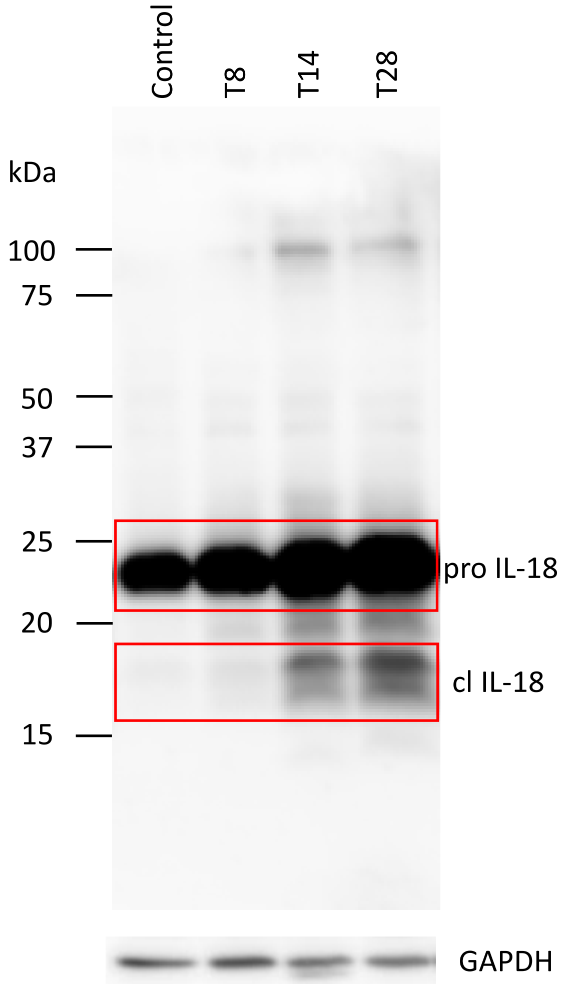

Supplemental Figure 3 (continued)

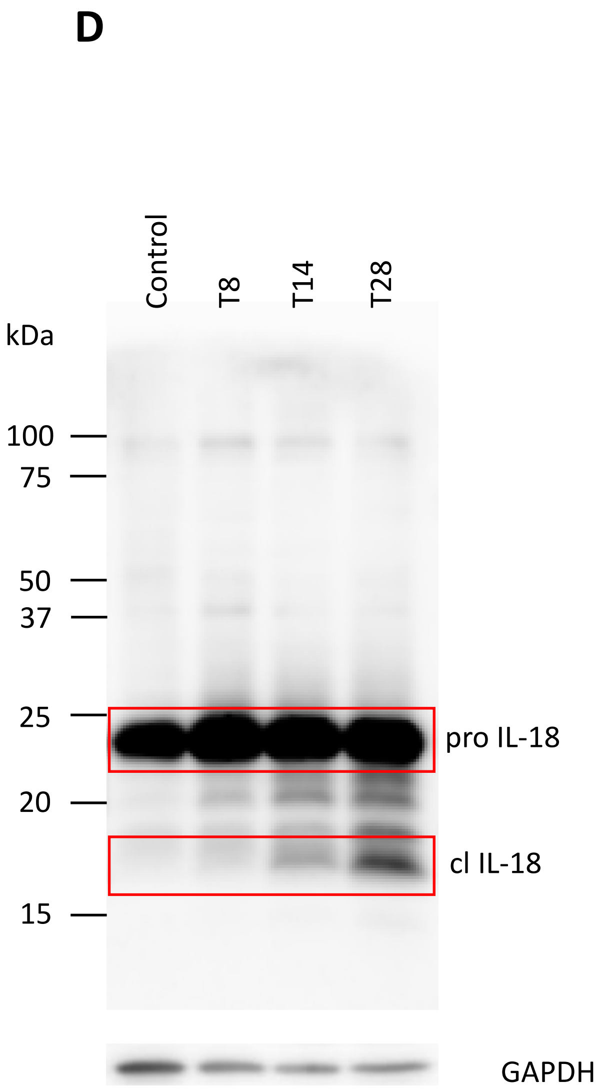

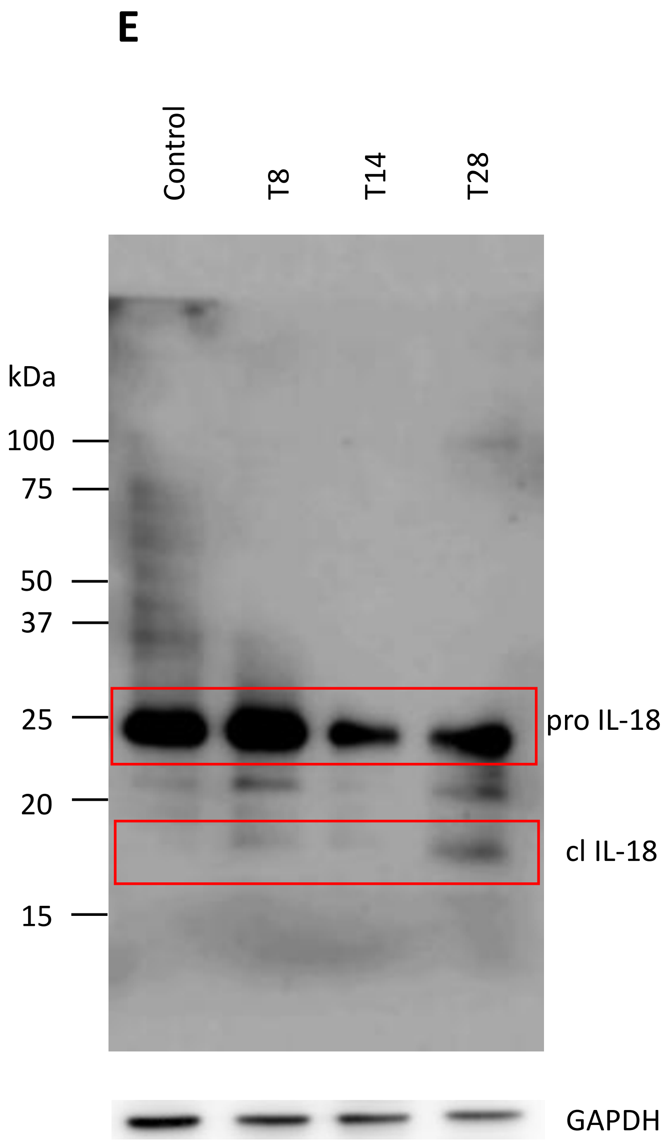

Supplemental Figure 3. Unedited Western blots of IL-18, used for quantification in figure 1. Parts of the Western blots used for quantification are boxed.

Supplemental Figure 4

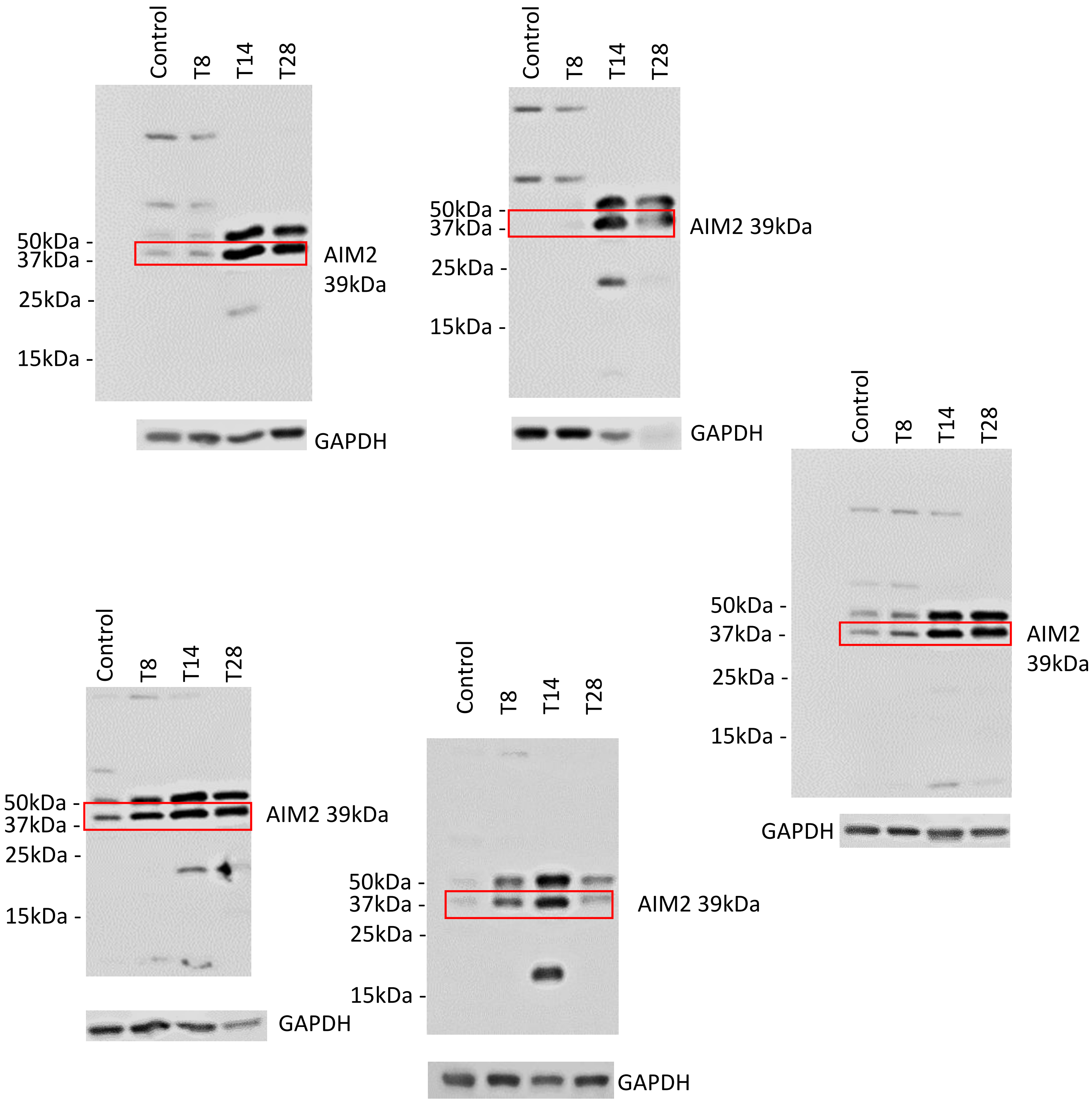

Supplemental Figure 4. Unedited Western blots of AIM2, used for figure 1. Parts of the Western blots used for quantification are boxed.

Supplemental Figure 5

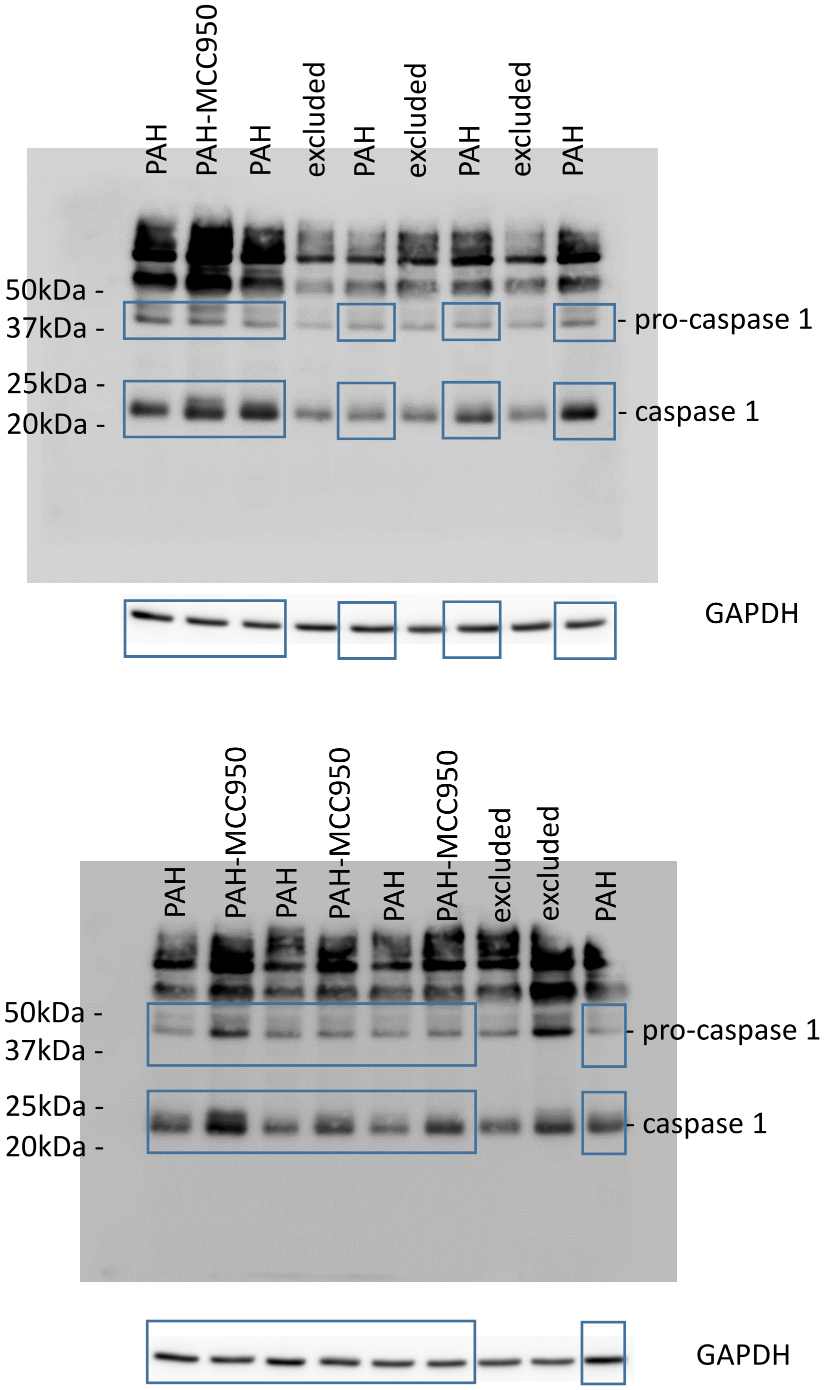

Supplemental Figure 5. Unedited Western blots of caspase-1, used for quantification in figure 2. Parts of the Western blots used for quantification are boxed. Four animals that received MCC950 and one that received vehicle were excluded in the analysis as described in the methods. This exclusion is indicated above the Western blot images for each animal separately.

Supplemental Figure 6

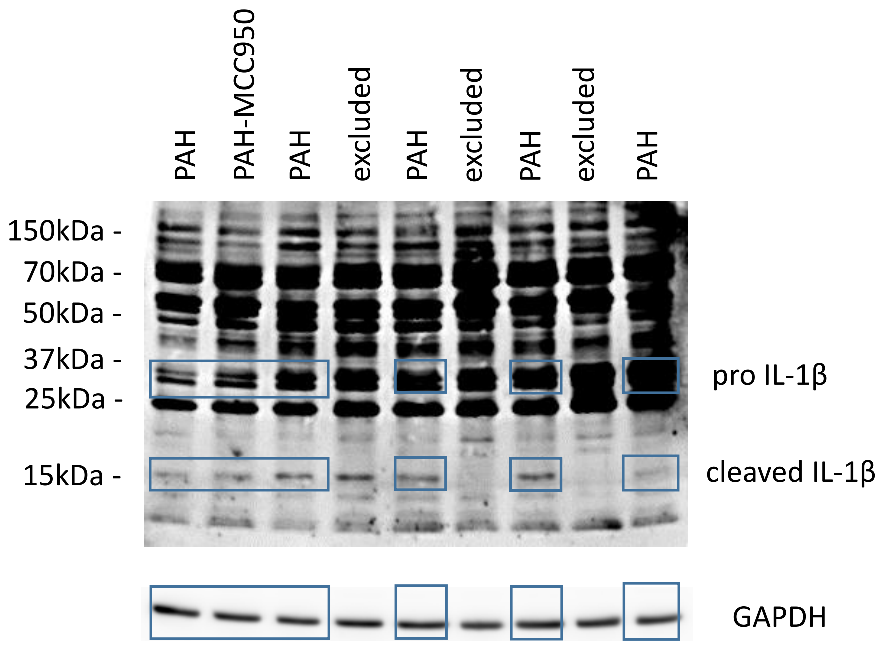

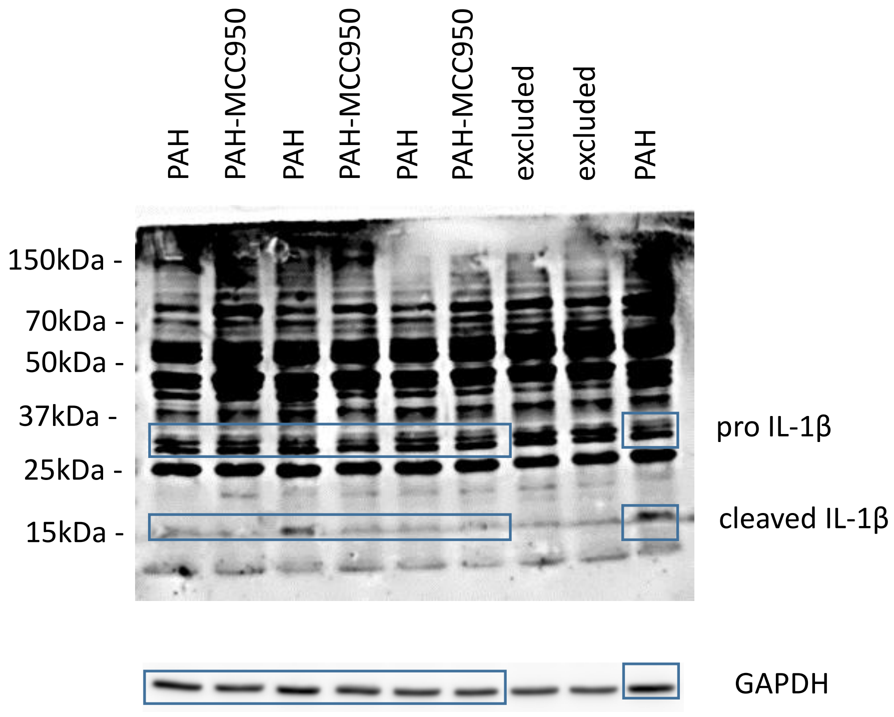

Supplemental Figure 6. Unedited Western blots of IL-1β, used for quantification in figure 2. Parts of the Western blots used for quantification are boxed. Four animals that received MCC950 and one that received vehicle were excluded in the analysis as described in the methods. This exclusion is indicated above the Western blot images for each animal separately.

Supplemental Figure 7

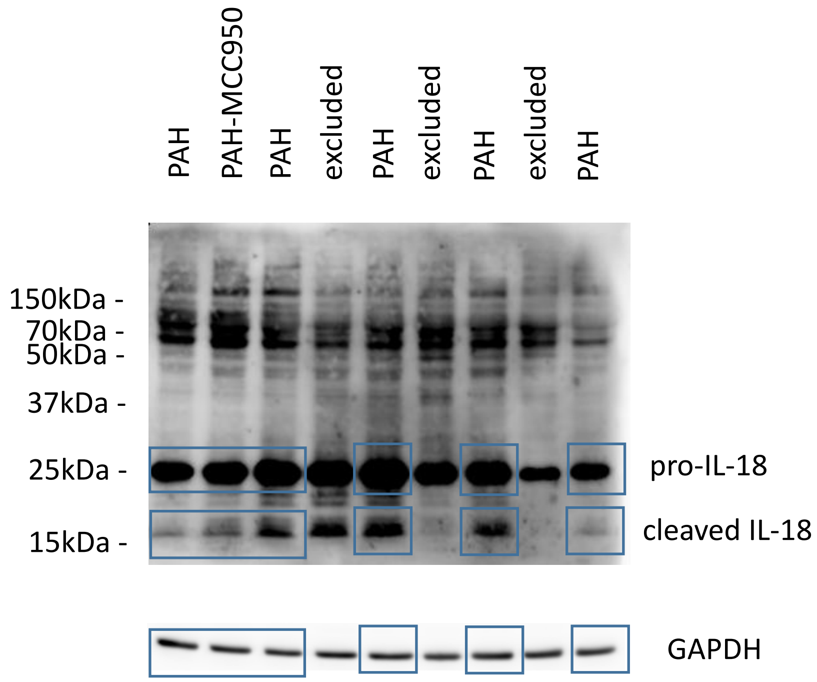

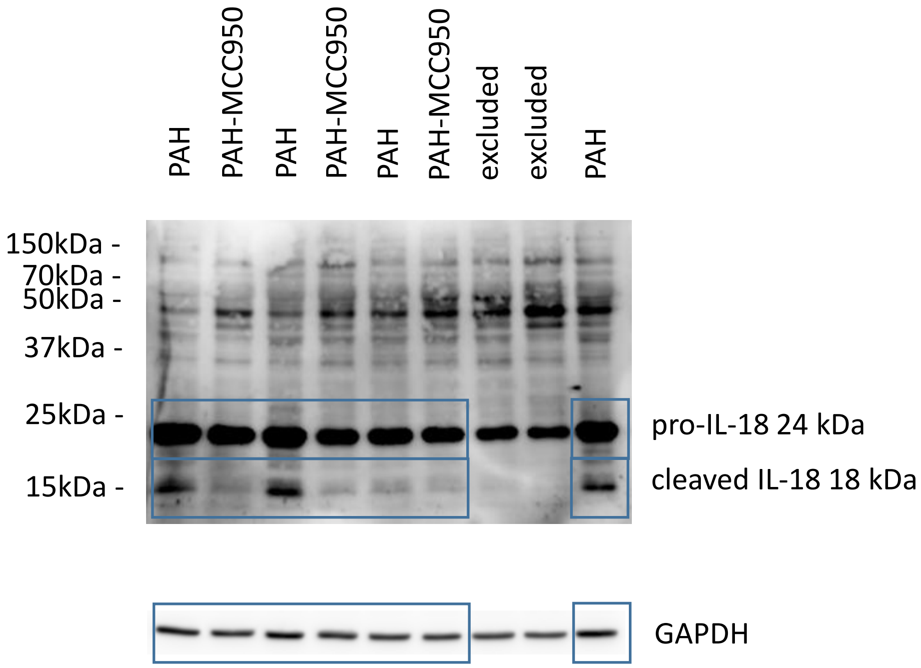

Supplemental Figure 7. Unedited Western blots of IL-18, used for quantification in figure 2. Parts of the Western blots used for quantification are highlighted. Four animals that received MCC950 and one that received vehicle were excluded in the analysis as described in the methods. This exclusion is indicated above the Western blot images for each animal separately.

Supplemental Figure 8

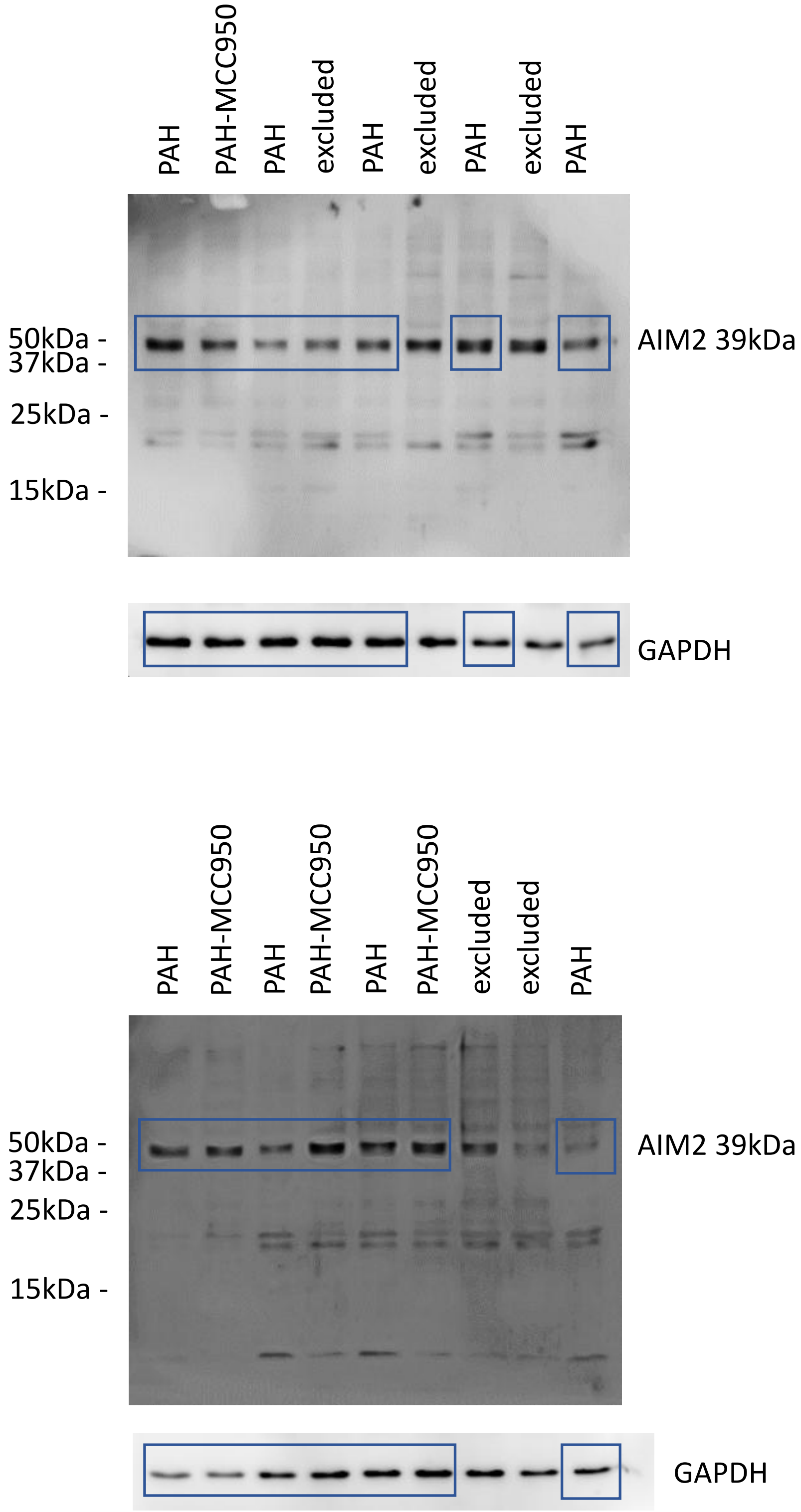

Supplemental Figure 8. Unedited Western blots of AIM2 for supplemental figure 10. Parts of the Western blots used for quantification are boxed. Four animals that received MCC950 and one that received vehicle were excluded in the analysis as described in the methods. This exclusion is indicated above the Western blot images for each animal separately.

Supplemental Figure 9

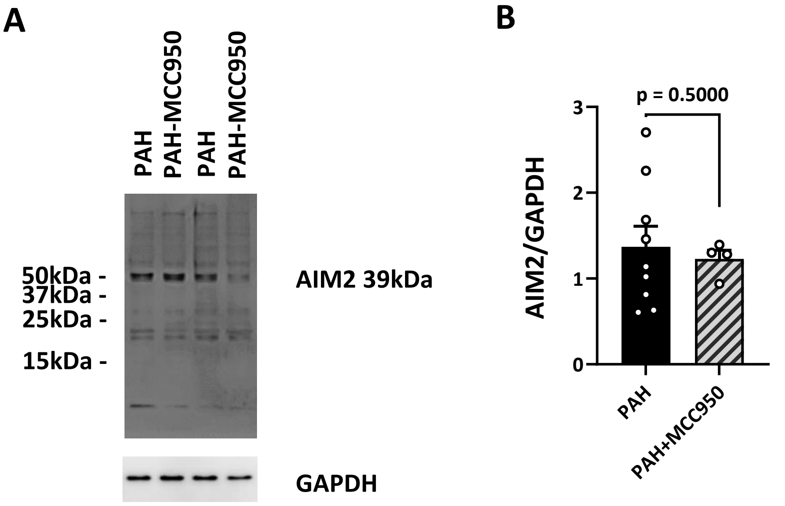

Supplementary Figure 9: Level of AIM2 in the lungs of the aortocaval shunt and monocrotaline rat model of PAH after treatment with MCC950. After aortocaval shunt surgery and monocrotaline injection (as described in the legends of Figure 1), PAH rats received MCC950 injections (10mg/kg in PBS daily, intraperitoneal) or vehicle (PBS injections, intraperitoneal) from the day after surgery onwards. On day 28, animals were sacrificed by exsanguination from the abdominal aorta and lungs were collected. Lung tissue was homogenized and AIM2 was assessed by Western blot. (**A**) Representative immunoblots. (**B**) Quantification of AIM2. Data are shown as mean ± SEM. The differences between the PAH and the PAH+MCC950 group were assessed using one-tailed Mann-Whitney test. Exact p-values are indicated on the graphs. PAH, pulmonary arterial hypertension.

Supplemental Figure 10

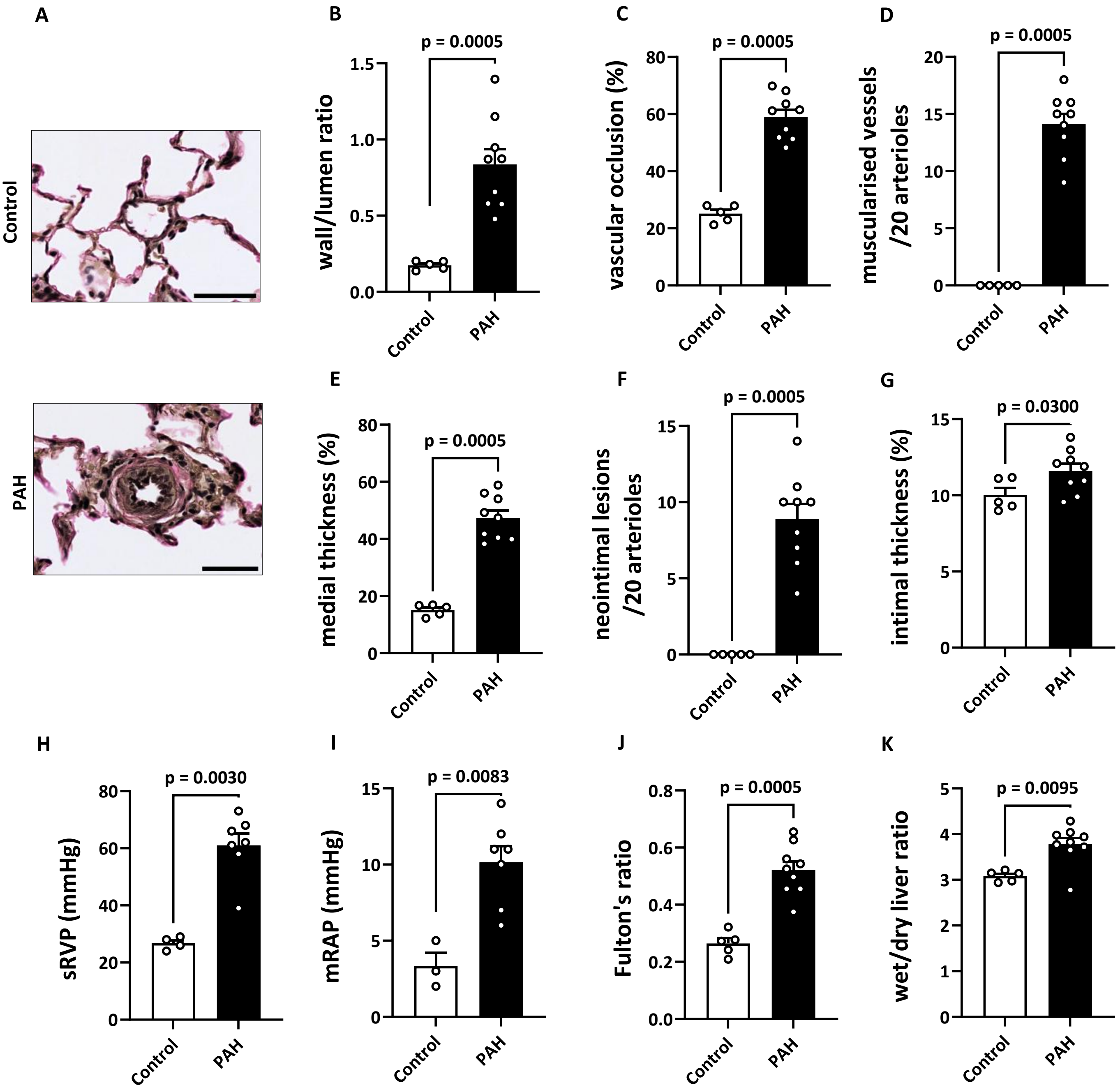

Supplementary Figure 10: Validation of PAH induction in the aortocaval shunt and monocrotaline rat model via pulmonary vascular remodelling and hemodynamics. Lungs collected 21 days after aortocaval shunt surgery were embedded in paraffin, sectioned (4 µm) and stained for Verhoeff's elastin. 20 pulmonary intra-acinar arterioles per animal were randomly selected for quantitative morphometric analyses. (**A**) Representative images of pulmonary intra-acinar arterioles in control and PAH rats. Scale bar: 50 µm. (**B-G**) Wall to lumen ratio, quantification of vascular occlusion, proportion of muscularised vessels, medial thickness, proportion neointimal lesions, and intimal thickness. (**H-K**) Hemodynamic parameters were assessed 21 days after aortocaval shunt surgery. Quantification of hemodynamic parameters included right ventricular systolic pressure (sRVP), mean right atrial pressure (mRAP), Fulton’s ratio and liver's wet to dry weight ratio. Data are shown as mean ± SEM. The Mann-Whitney test was performed to detect differences between groups. Exact P-values (where P<0.05) are indicated on the graphs.

Supplemental Figure 11

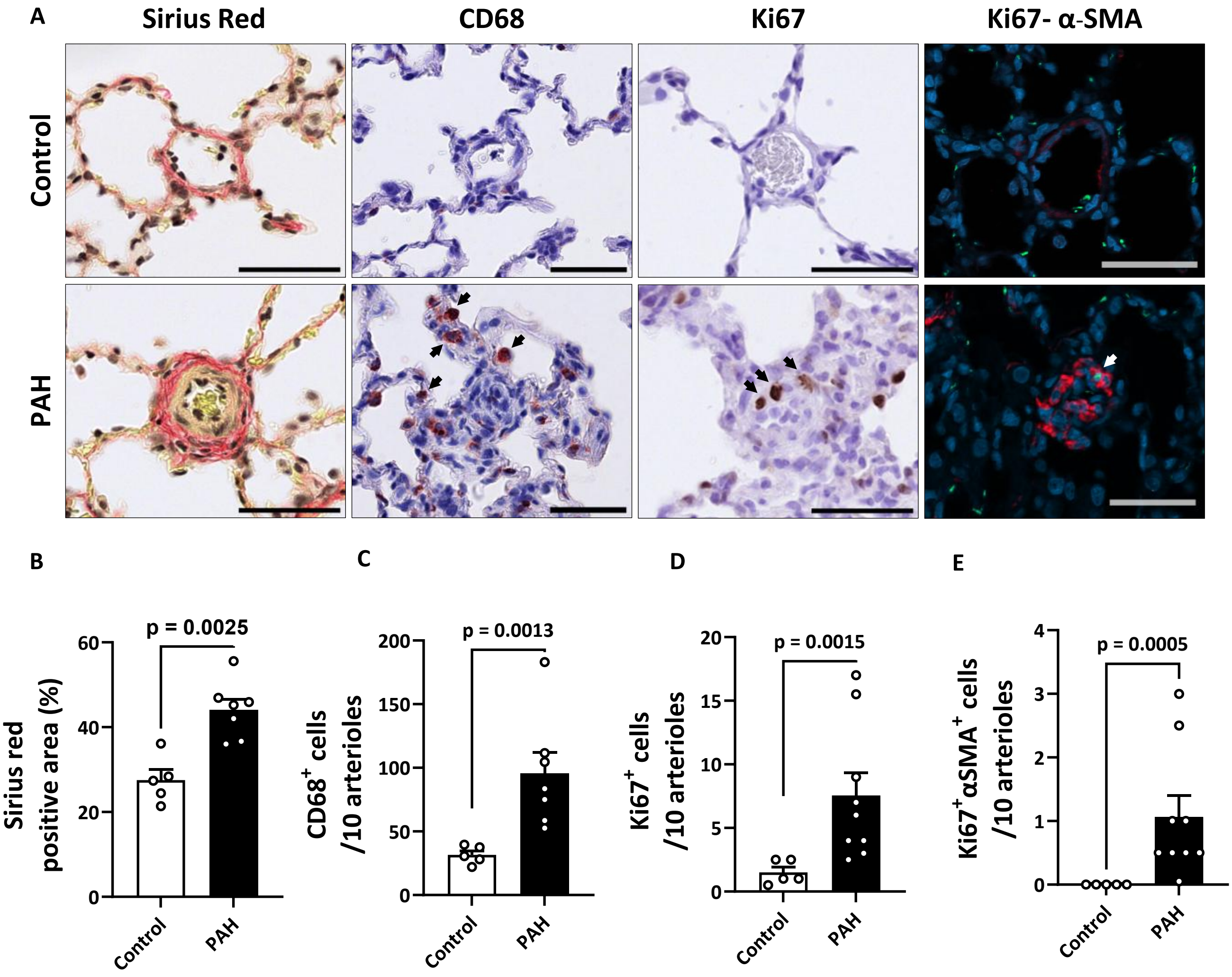

Supplementary Figure 11: Collagen deposition, macrophage infiltration and cell proliferation in the control and the PAH group. (**A**) Representative images for Sirius Red (collagen staining), CD68 (macrophage marker), Ki67 (proliferation marker), and dual IF staining for Ki67 and α-SMA (smooth muscle cell marker), in control and PAH groups, with $CD68^+$ perivascular and transmural cells indicated by black arrows (middle, left), $Ki67^+$ cells in the vascular wall of intra-acinar vessels indicated by black arrows (middle, right), and the overlay between $Ki67^+$ cells (green) and α-SMA (red) indicated by white arrows (right); (**B**) Sirius Red staining was quantified as mean collagen positive area from 20 randomly selected pulmonary intra-acinar vessels (< 50 µm diameter) per rat. (**C**) CD68 was quantified as the mean number of positive cells in 20 vessels per rat. Cells were counted within a ROI 50 µm from the outer vessel border. (**D**) $Ki67^+$ cells were quantified as the mean number of positive cells in 20 vessels per rat. (**E**). Overlay of Ki67+ and, α-SMA cells within the vascular wall for PAH and PAH+MCC950 groups. Two animals were excluded from the PAH group for the analysis due to collapsed lung tissue. The data are shown as mean ± SEM and were analyzed by one-tailed Mann Whitney test. Scale bar: 50µm.

**Supplemental Table 1: Specifications of antibodies for immunohistochemical and immunofluorescent stainings**						
Antibody	Species	Company	Catalog nr	Dilution	Experiment/Tissue	Secondary antibody (if applicable)
anti-NLRP3	rabbit	Novus Biologicals	NBP2-12446	1:100 (for rats)<br>1:50 (for human)	• IF rat lungs<br>• IF Human LTx	• Goat anti-Rabbit IgG (H+L), Alexa Fluor 488, – 1:100<br>• Goat anti-Rabbit green, Alexa Fluor 488 highly cross absorbed (#a-11034, Thermo Fisher Scientific) – 1:100
anti-AIM2	rabbit	Thermo Fisher Scientific	14-6008-93	1:100 (for rats)<br>1:250 (for human)	• IF rat lungs<br>• IF Human LTx	• same as for anti-NLRP3
anti-CD68	mouse	Bio-Rad	MCA341GA	1:100	• IF rat lungs<br>• IHC rat lungs	• IgG Goat anti-Mouse Alexa Fluor 647 Ab150115, Abcam - 1:100<br>• Horse anti-mouse IgG Biotinylated (BA 9500, Vector Laboratories) – 1:125
anti-CD68	mouse	Thermo Fisher Scientific	MA5-13324	1:100	IF Human LTx	Goat anti-Mouse Alexa Fluor 647, Ab150115, Abcam – 1:100
anti-Ki67	rabbit	Thermo Fisher Scientific	RM-9106-S0	1:50	• IF rat lungs<br>• IHC rat lungs	• Goat anti-Rabbit IgG (AF488), H&L, #A11034, Thermo Fisher – 1:200<br>• Goat anti-Rabbit biotinylated, BA-1000, Vector, 1:250 combined with ABC complex, PK-4000, Vector
anti-SMA	mouse	Proteintech	67735-1-Ig	1:100	IF rat lungs	Goat Anti-Mouse IgG H&L (AF647), Ab150115, Abcam – 1: 200
